# Split versions of *Cleave and Rescue* selfish genetic elements for measured self limiting gene drive

**DOI:** 10.1371/journal.pgen.1009385

**Published:** 2021-02-18

**Authors:** Georg Oberhofer, Tobin Ivy, Bruce A. Hay

**Affiliations:** California Institute of Technology, Pasadena, California, United States of America; Fred Hutchinson Cancer Research Center, UNITED STATES

## Abstract

Gene drive elements promote the spread of linked traits, providing methods for changing the composition or fate of wild populations. Drive mechanisms that are self-limiting are attractive because they allow control over the duration and extent of trait spread in time and space, and are reversible through natural selection as drive wanes. Self-sustaining *Cleave and Rescue* (*ClvR*) elements include a DNA sequence-modifying enzyme such as Cas9/gRNAs that disrupts endogenous versions of an essential gene, a tightly linked recoded version of the essential gene resistant to cleavage (the *Rescue*), and a Cargo. *ClvR* spreads by creating loss-of-function (LOF) conditions in which those without *ClvR* die because they lack functional copies of the essential gene. We use modeling to show that when the *Rescue*-Cargo and one or both components required for LOF allele creation (Cas9 and gRNA) reside at different locations (split *ClvR*), drive of *Rescue*-Cargo is self-limiting due to a progressive decrease in Cas9 frequency, and thus opportunities for creation of LOF alleles, as spread occurs. Importantly, drive strength and duration can be extended in a measured manner—which is still self-limiting—by moving the two components close enough to each other that they experience some degree of linkage. With linkage, Cas9 transiently experiences drive by hitchhiking with *Rescue*-Cargo until linkage disequilibrium between the two disappears, a function of recombination frequency and number of generations, creating a novel point of control. We implement split *ClvR* in *Drosophila*, with key elements on different chromosomes. Cargo/*Rescue*/gRNAs spreads to high frequency in a Cas9-dependent manner, while the frequency of Cas9 decreases. These observations show that measured, transient drive, coupled with a loss of future drive potential, can be achieved using the simple toolkit that make up *ClvR* elements—Cas9 and gRNAs and a *Rescue*/Cargo.

## Author summary

There is great interest in being able to spread beneficial traits throughout wild populations. However, many traits of interest do not provide a fitness benefit to those who carry them, and thus require some kind of a “push” to drive them to high frequency in the population. Selfish genetic elements are transmitted to viable, fertile progeny at rates greater than those of other genes, and can spread to high frequency even when they result in fitness costs to carriers—a phenomenon known as gene drive. If genes encoding traits of interest are incorporated into a selfish genetic element, they too can be forced to spread. Gene drive methods that provide a driving force that ultimately wanes to nothing over time (that are self-limiting) are attractive because they ensure that spread to high frequency will be limited in time and space, features that are likely to be important in many regulatory and social environments. Here we use modeling and experiments in *Drosophila melanogaster* to show how split versions of a gene drive method known as *Cleave and Rescue* (*ClvR*) can be used to bring about self-limiting drive whose strength and duration can be tailored to specific economic, ecological and/or regulatory needs.

## Introduction

Gene drive occurs when specific alleles are transmitted to viable, fertile progeny at rates greater than those of competing allelic variants. When alleles of genes conferring traits of interest are linked with a synthetic genetic element that mediates self-sustaining drive, spread to high frequency in otherwise wildtype (WT) populations can be achieved for population modification [1–8] and population suppression [9–11], forms of genetic population management. These drive mechanisms must be strong enough to spread to high frequency on human timescales, but must also function within diverse and evolving social and regulatory frameworks (reviewed in [12,13]). Central to these considerations are issues of confinement and reversibility: can the spread of transgenes to high frequency be limited to locations in which their presence is sought; can drive be terminated; can new population modifications be exchanged for old ones; and can the population be restored to the pre-transgenic state? Given the diversity of possible scenarios in which drive is being considered, and the competing mandates that any transgene-based technology meant for implementation in the wider world must contend with, no one drive method will be suitable for all occasions, or perhaps even be ideally suited to any particular occasion. Thus, as exploration continues into how gene drive can best be used in the wild it is important to have (in forms that can plausibly be implemented) a diversity of gene drive methods with different characteristics in terms of cost to initiate and maintain a modification at high frequency in a target population, and to confine, terminate, modify or reverse these modifications.

Low threshold self-sustaining gene drive mechanisms include homing and Y-drive. These are are strong drivers at low frequency and in the presence of significant fitness costs [14,15]. *Medea* [1,16,17] *Cleave and Rescue* (*ClvR*) [5,6], TARE [18] and a number of other proposed gene drive elements [19,20] are also low threshold. However, these are weak drivers at low frequency and acquire a threshold in the presence of any fitness costs. All are predicted to be relatively invasive and may (depending on the details of the system and the ecology) be challenging to confine to a target area due to the fact that continuous low level migration of drive element-bearing individuals into neighboring areas can result in these areas being seeded with enough transgene-bearing individuals that spread to high frequency occurs [15,21–25].

High threshold self-sustaining gene drive mechanisms include various forms of engineered single- or multi-locus toxin-antidote systems [3,19,20,26–30], and chromosome rearrangements such as translocations, inversions and compound chromosomes [4,31,32,33]. These drive using the phenomenon of frequency-dependent underdominance. They require that transgenes make up a much larger fraction of the total wild population before gene drive occurs. Below this frequency transgenes are actively eliminated from the population. High threshold mechanisms are more confinable than low threshold mechanisms by virtue of the fact that the threshold frequency needed for drive in neighboring non-target populations is—depending on the details of the system and migration rate—less likely to be achieved [25,34–40]. Transgenes can also in principle be eliminated from the population if release of WT results in the frequency of transgenics being driven below the threshold required for drive. However, modeling shows that the ability of high threshold drive elements to spread to high frequency in a target area––versus being pushed out of it––while avoiding spread to high frequency in neighboring regions, depends on the details of the drive system, and key aspects of the local ecology, such as migration rates, dispersal distance, and the density and fitness of different genotypes in border regions (reviewed in [41]). These variables may often be difficult to quantify, resulting in uncertainty as to whether a drive element implemented in a specific species will spread throughout a target region (versus being eliminated), and remain restricted to that region, in any particular environment.

Given the challenges––in contexts in which the drive is not simply meant to spread to genotype (all are transgene carriers) or allele (all alleles are transgenic) fixation throughout the species range––associated with regulating the behavior and fate of self-sustaining drive elements, there is also interest in a second family of approaches, which are our focus here. These include a component of gene drive––which can be regularly reinforced through continued releases––but are also guaranteed by virtue of the genetics associated with their mechanism of action to lose drive potential (the ability to spread a linked Cargo) at a predictable rate. Non-self sustaining (self-limiting) drive mechanisms are attractive because spread of the desired trait is ultimately always limited in time and space, regardless of the presence or absence of specific physical or ecological barriers or levels of migration. Several such systems have been proposed. These include various forms of split homing endonuclease genes (a split-HEG) [37,42–56], and *Killer-Rescue* [57,58]. Y-linked genome editors coupled with an X shredder have also been proposed for self-limiting drive, but are designed to bring about populations suppression [59]. Each works by dividing the gene drive element into two physically separate components, one that is driven into the population and is tightly linked to Cargo genes (endogenous alleles or transgenes that use linkage to hitchhike to high frequency), and another (which is typically genetically unlinked, though see below) that brings about drive (the driver element), but that has no (or only a transient) ability to enhance its own rate of transmission. There is a progressive decrease in population frequency of the driver element as spread of the Cargo-linked element (the element that is driven) occurs. This results from the dispersal of individuals, dilution by WT, and loss by natural selection. The resultant loss of drive activity ultimately limits the spread of Cargo regardless of other variables. The fate of the Cargo is then dependent on the rate at which it is eliminated from the population through natural selection, a process that can potentially be enhanced through the incorporation of unconditional or environmental condition-dependent fitness costs into the Cargo-bearing element.

A simple split HEG locates gRNAs (and any other Cargo genes) at the site of cleavage and homing (thereby disrupting the target sequence), while Cas9 is located elsewhere in the genome. In this configuration a homing based increase in gRNA/Cargo copy number only occurs when Cas9 activity and the gRNA/Cargo cassette are present in the same individual. Daisy drive uses a similar strategy, but it is stronger and more invasive in neighboring populations because it includes multiple layers of homing [37,42,43]. Most recently, other configurations have been modeled that utilize homing coupled with Killer and *Rescue*-based mechanisms (split-drive *Killer-Rescue* (SDKR) [44], or other implementations of underdominance [45,46], some of which can be self-limiting or self sustaining. Split HEGs have been created in *Drosophila* [47–54] and mosquitoes [55,56], but not yet shown to spread to high frequency in otherwise WT populations (populations in which Cas9 is not already made ubiquitous through prior population engineering), Implementations of Daisy drive and SDKR have not yet been described.

In the *Killer-Rescue* system [57] there are two unlinked genes, a zygotic toxin (the *Killer*), which serves as the driver, and a zygotic antidote (the *Rescue*), to which Cargo transgenes are tightly linked (below we refer to this system as *Killer-Rescue*/Cargo). When individuals bearing *Killer* and *Rescue*/Cargo (which are unlinked) are released into a WT population, progeny that inherit the *Rescue*/Cargo with or without the *Killer* survive, while those that inherit the *Killer* but not the *Rescue*/Cargo die. This latter activity serves to bring about an increase in the frequency of the *Rescue*/Cargo-bearing chromosome relative to its WT counterpart. Levels of the *Killer* drop over time whenever it finds itself in non-*Rescue*/Cargo-bearing individuals, and in response to natural selection acting on any associated fitness costs. As the frequency of the *Killer* fades, so too does the drive that maintains the *Rescue*/Cargo construct in the population. An implementation of *Killer-Rescue* has recently been described in *Drosophila*, showing that (with some tinkering, required to identify *Killers* and *Rescues* that worked well together) self-limiting drive can be successful [58].

Finally, Y-linked genome editors coupled with a second element that shreds the X chromosome provide another strategy for self-limiting drive, in this case designed to bring about population suppression [59]. A Y-linked genome editor cleaves or otherwise modifies an X-linked locus (such as a haplolethal) to bring about dominant lethality of female progeny (because only female progeny inherit the X from the male). The transgene-bearing Y chromosome does not suffer this cost, but does not drive since there is no selection against WT Y chromosomes. An autosomal (or X-linked) X shredder cleaves the X chromosome during spermatogenesis, resulting (hopefully) in a male progeny bias due to loss of X-bearing sperm. This also does not drive since the transgene-bearing chromosome finds itself in X-bearing sperm half the time. However, when the two elements are combined, the X shredder temporarily drives the Y-linked genome editor to higher frequency, augmenting its ability to suppress the population in a self limiting manner. The possibility of using linkage between these components, when present in the X-Y pseudoautsomal region, to extend drive lifetime in a self-limiting manner, was noted but not explored in more detail.

Here we describe and implement a novel mechanism for self-limiting population modification drive, split *Cleave and Rescue* (split *ClvR*). Drive with split *ClvR* is much stronger (it drives transgenes with or without fitness costs to a higher frequency and for longer duration per unit introduction percent of transgene-bearing individual) than with *Killer-Rescue*. In addition, drive strength, and duration and extent in time and space for a given introduction percent can be extended in a measured manner—which is still self-limiting—simply by moving the two components close enough to each other on the same chromosome so that they experience some degree of linkage (segregate from each other during meiosis when placed in cis at rates <50%; a map distance of less than 50 centiMorgans, cM). Finally, we describe an implementation of split *ClvR* in *Drosophila* and show that when it is introduced into an otherwise WT population, *Rescue*/Cargo spreads to high frequency while the frequency of the Cas9, which is required for drive, decreases. Introduction of WT into this modified population results in no further drive, thereby demonstrating that drive is transient.

## Results

### Basis for gene drive by *ClvR* elements

**Fig 1:**
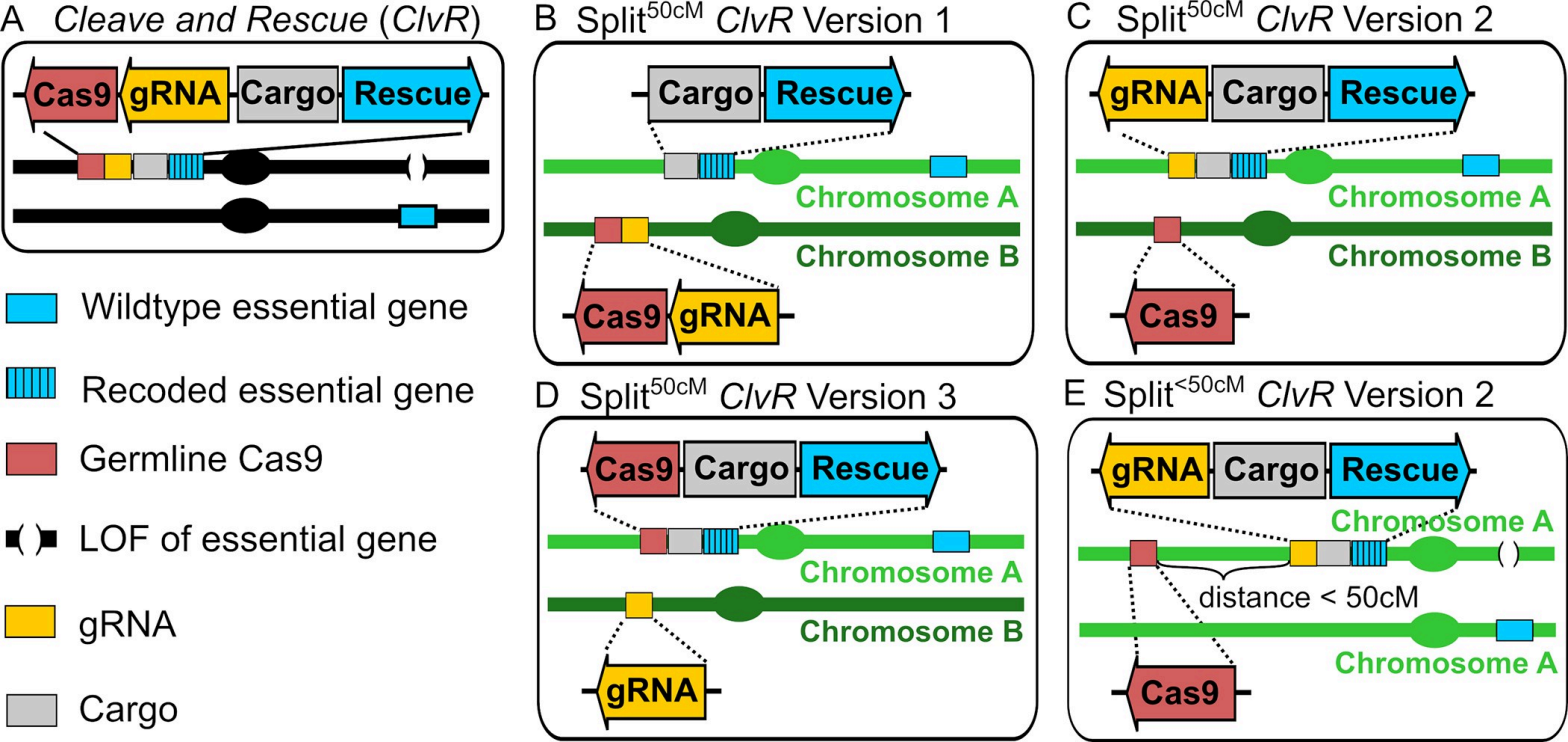
*ClvR* and split *ClvR* configurations. Split *ClvR* with independent segregation (split^50cM^ *ClvR*) can be implemented in multiple configurations, each of which leads to drive of Rescue/Cargo to higher frequency. **(A)** *ClvR*. **(B)** Split *ClvR*^50cM^ Version 1, in which Cargo/*Rescue* and Cas9/gRNAs are on separate chromosomes, and thus show independent segregation (map distance of 50 cM (centi Morgan)) during meiosis. **(C)** Split^50cM^ *ClvR* Version 2, in which Cas9 is on a different chromosome from Rescue/Cargo/gRNAs. **(D)** Split*ClvR*^50cM^ Version 3, in which gRNAs are on a different chromosome from Cas9/Cargo/*Rescue*. **(E)** Split *ClvR*^<50cM^ Version 2, in which Cas9 is located on the same chromosome as Rescue/Cargo/gRNAs, at a distance of less than 50 cM. Other versions of split *ClvR* (Version 1 and Version 3), in which all components are on the same chromosome at a distance of less than 50 cM are shown in S1 Fig.

A *ClvR* element [5,6]) (also known as Toxin Antidote Recessive Embryo (TARE) in a related proof-of-principle implementation [18]), which serves as the starting point for this work, is a self-sustaining gene drive element (Fig 1A). It consists of a DNA sequence-modifying enzyme such as Cas9/gRNAs that disrupts endogenous versions of an essential gene (located anywhere in the genome) in the germline and in the zygote using Cas9/gRNAs carried over from the mother, and a tightly linked version of the essential gene recoded to be resistant to cleavage and ectopic gene conversion with the endogenous locus (the *Rescue*) [5,6,18]. *ClvR/TARE* (hereafter referred to as *ClvR*) spreads because Cas9/gRNAs create loss-of-function (LOF) alleles (the drive force) that select against those who fail to inherit *ClvR* in LOF homozygotes. In contrast, those who inherit *ClvR* always survive (in the case of a haplosufficient gene) because they inherit the *Rescue* transgene, which is tightly linked to one or more Cargo genes (*Rescue*/Cargo). Gene drive with *ClvR* is self sustaining because tight linkage between Cas9/gRNAs (the driver: that which creates the drive force) and *Rescue/*Cargo (the component being driven) results in both sets of components continuously experiencing the drive benefits of LOF allele creation––an increase in frequency relative to that of the non-*ClvR* chromosome. Drive with *ClvR* is frequency-dependent, slow and weak at low frequencies, and rapid and strong at higher frequencies. It lacks a release threshold when fitness costs are absent, but acquires one in their presence. When drive occurs, transgenes spread to genotype or allele fixation depending on the location of *ClvR* and the gene being targeted [5,6,18,20]. Finally, haploinsufficient or haplolethal genes can also be targeted. Higher thresholds for drive are created, but when drive occurs, it still leads to transgene and/or allele fixation [5,6,18,20].

### Split versions of *ClvR* locate the *Rescue*/Cargo and one or both components of Cas9/gRNAs at different genomic positions

We now consider cases in which the *Rescue*/Cargo and one or both components of germline-expressed Cas9/gRNAs are located on different chromosomes, and thus segregate independently of each other (50 cM apart), creating versions of split *ClvR* designated as split^50cM^ *ClvR*. Three configurations are possible. In version 1 (split^50cM^ *ClvR* V1) the Cas9/gRNA construct is located on a different chromosome from that of the Rescue/Cargo (Fig 1B). In versions 2 and 3 (Fig 1C and 1D) only one of the Cas9/gRNA components is located on a different chromosome. Because these latter two behave identically, we focus below on split^50cM^ *ClvR* V2. With split^50cM^ *ClvR* V1, individuals carrying only the Cas9/gRNA-bearing construct experience essential gene cleavage and LOF allele creation, resulting in death or sterility (and loss of Cas9-bearing and non-*Rescue*/Cargo chromosomes). In contrast, with split^50cM^ *ClvR* V2 cleavage and LOF allele creation only occurs when *Rescue*/Cargo/gRNA and Cas9 find themselves in the same (viable and fertile) individual. Split^50cM^ *ClvR* V2 (implemented in the experimental section below) is particularly easy to synthesize since organisms carrying each transgene-bearing component are homozygous viable in isolation. In contrast, for split^50cM^ *ClvR* V1 the Cas9/gRNA transgenes must be introduced into, and maintained within the *Rescue*/Cargo background, as with *ClvR* [5,6]. Versions of split *ClvR* in which there is linkage between the components (Fig 1E, genetic map distance <50cM on the same chromosome) are discussed in a later section.

### Features of the model used to characterize Split *ClvR*

We model the behavior of split *ClvR* using a commonly used framework [2,5,6,19,37,57,60,61] that assumes random mating, non-overlapping generations with no age structure, infinite population size (deterministic populations), and equal and additive fitness costs for each of the transgenes. We characterize results from a set of scenarios that illuminate general features of split *ClvR* drive rather than an exhaustive analysis of all possible conditions. We use *ClvR* and *Killer-Rescue*/*Cargo* as points of comparison with split *ClvR* since each utilizes a toxin-antidote mechanism of action, and *ClvR* [5,6,18] and *Killer-Rescue*/Cargo [58] have been successfully implemented. In general the plots below show genotype frequencies (the frequency of transgene carriers). If a plot shows allele frequencies this is indicated specifically. Finally, our focus is primarily on versions of split *ClvR* in which the *Rescue*/Cargo locus is different from that of the essential gene being targeted (see Fig 1). The behavior of versions of split *ClvR* in which *Rescue*/Cargo and the essential gene are at the same locus are discussed briefly below. Finally, for these and other experiments we sometimes use time frames of 300 generations or more to capture the long-term dynamics intrinsic to the genetics of the split *ClvR* drive system. However, it should be understood that mutation of Cargo, drive components and/or mutations or polymorphisms in target sites, particularly when these result in reduced fitness costs to carriers, will often result in reduced *effective* lifetimes of *Rescue*/Cargo at high frequency (see analysis in examples from several different systems [5,6,18,23]). Finally, since in deterministic populations genotypes and alleles can never reach fixation, here and below the phrase “approaches fixation” refers to cases in which an allele or genotype has a prolonged residence time at frequencies >99%. Model code can be found in the Hay Lab Split-ClvR repository at https://github.com/HayLab/Split-ClvR. An extended version of this model will be described in more detail elsewhere.

### Split *ClvR* with independent segregation (split^50cM^ *ClvR*) provides strong, transient drive that is driven by the creation of LOF alleles at the essential gene locus

**Fig 2:**
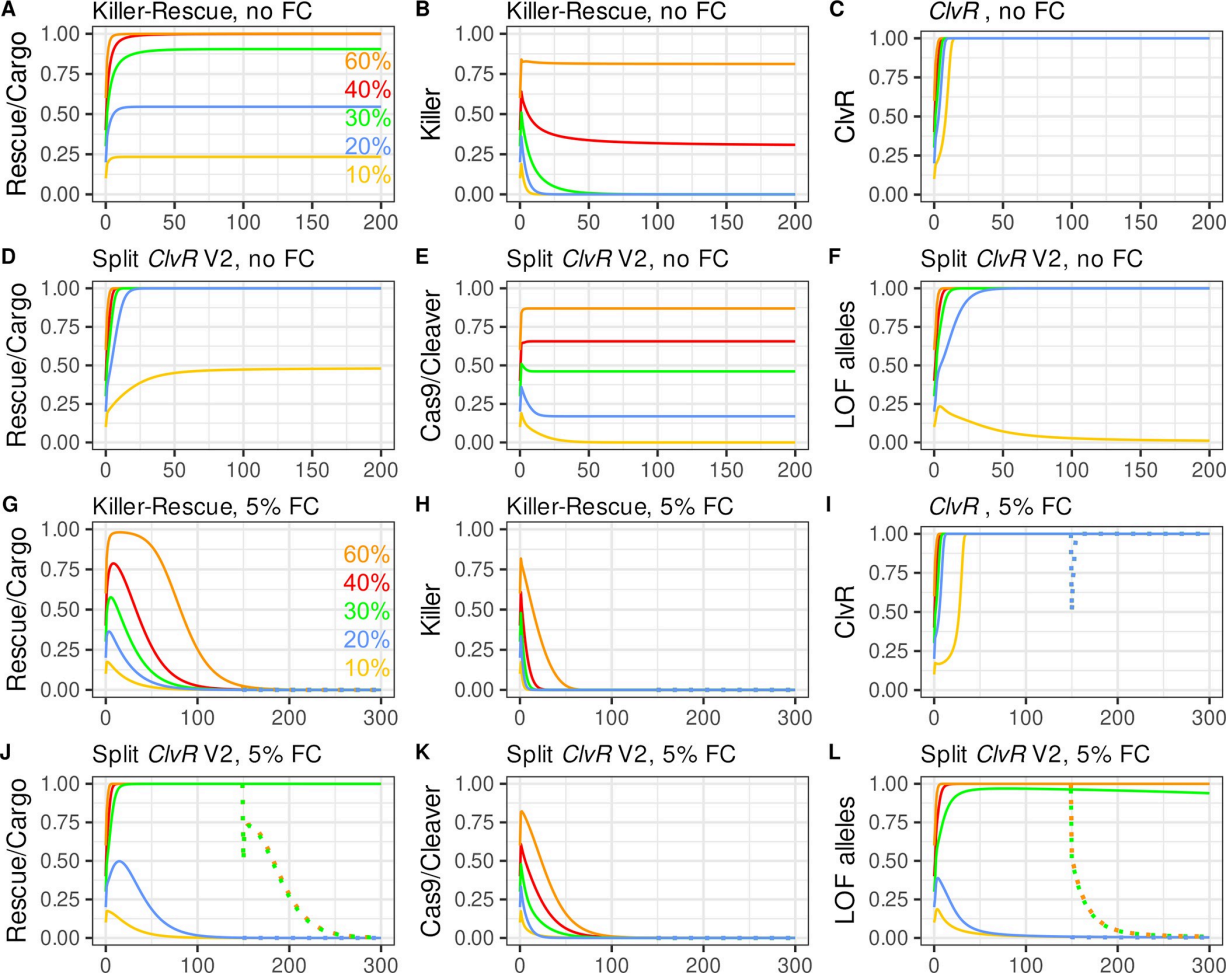
Population dynamics of *Killer-Rescue*/Cargo, *ClvR* and split^50cM^ *ClvR* V2. Drive with split *ClvR* with independent segregation (split^<50cM^ *ClvR*) is stronger than that of *Killer-Rescue* in the absence or presence of fitness costs. **(A-F, and J-O)** Plotted are genotype frequencies for *Rescue*/Cargo and Cas9, and allele frequencies for LOF (y-axis) over generations (x-axis). Release percentages are 10% (yellow), 20% (blue), 30% (green), 40% (red), and 60% (orange). **(A-F)** elements have no fitness costs; **(G-L)** 5% additive fitness cost/allele. In panels G-L there is a 50% release percentage of WT in generation 150 (dotted lines).

We begin by showing drive behavior for *Killer-Rescue*/Cargo, *ClvR* and versions of split *ClvR*, when introduced at various frequencies, for elements that have no associated fitness costs. Except as noted below, with split *ClvR* we focus on implementations in which the *Rescue*/Cargo is located at a site distinct from that of the essential gene being targeted for LOF allele creation (distant site split *ClvR*). When the introduction percent of *Killer-Rescue*/Cargo is low (10–20%), killing of individuals lacking the *Rescue*/Cargo chromosome (which necessarily results in a loss of at least one *Killer* allele), leads to a transient increase in the frequency of the *Rescue*/Cargo-bearing chromosome (Fig 2A), but the *Killer* is lost before all non-*Rescue*/Cargo chromosomes have been eliminated (Fig 2B). This results in the frequency of the *Rescue*/Cargo plateauing at some level below allele fixation. At higher introduction percentages, levels of the *Killer* are sufficient to bring about the loss of essentially all non-*Rescue*/Cargo chromosomes. Remaining *Killer* alleles are now protected from loss and float in the population indefinitely, as described previously [57]. In contrast, with *ClvR*, *Rescue*/Cargo and Cas9/gRNAs spread rapidly to transgene fixation for all introduction percentages because tight linkage of Cas9/gRNAs to *Rescue*/Cargo protects the former from removal in LOF homozygotes and allows it to hitchhike with Cargo/*Rescue* to high frequency, thereby maintaining drive potential indefinitely (Fig 2C and [5,6,18].

Both versions of split^50cM^ *ClvR* show an intermediate behavior. Below we present the behavior of Version 2 (Fig 2D–2F). S2 Fig shows the behavior of Version 1, which is similar, though not identical. As with *ClvR*, drive of the *Rescue*/Cargo/gRNAs chromosome is mediated by the removal of non-*Rescue/*Cargo/gRNAs chromosomes in LOF homozygotes. In the case of split^50cM^ *ClvR* V1, the driver (Cas9/gRNAs) chromosome brings about the loss of non-*Rescue*/Cargo/gRNAs chromosomes and itself (in sterile or dead individuals) when it creates LOF alleles in the germ cells of those who carry it, and the driver chromosome is not protected by the presence of the *Rescue*/Cargo/gRNAs. This component of split^50cM^ *ClvR* V1 drive represents the limit of killing and drive by *Killer* in the *Killer-Rescue*/Cargo system. In contrast, with split^50cM^ *ClvR* V2, LOF allele creation only occurs when the two components are brought together in the same (viable and fertile) individual.

Importantly, with both versions of split^50cM^ *ClvR*, when Cas9/gRNAs or Cas9 are in a *Rescue*/Cargo or *Rescue*/Cargo/gRNAs background, respectively, not only are they protected from loss (as with *Killer-Rescue*/Cargo), they still create the LOF alleles at the third, essential gene locus that mediate drive (Fig 2F). With this “action at a distance”, split^50cM^ *ClvR* elements create a powerful drive force that can manifest itself in future generations, in individuals that need not carry the driver chromosome (see S2G–S2I and S3 Figs for examples.). In short, while LOF alleles that mediate drive require Cas9 endonuclease activity for their creation, they exist and segregate independently of it, and do not require its presence (or its loss) for their killing activity. Equally important, the increase in frequency of *Rescue*/Cargo/gRNAs brought about by removal of non-*Rescue*/Cargo/gRNAs chromosomes in LOF homozygotes works (with the distant site split *ClvR* being discussed here) to promote the maintenance of LOF alleles in the population because as the frequency of *Rescue*/Cargo/gRNAs increases, the selection pressure that would otherwise bring about the removal of LOF alleles in homozygotes decreases. In consequence, in populations in which *Rescue*/Cargo/gRNAs has been driven to high frequency, LOF alleles can be maintained at high frequency as a latent drive force that manifests *only* when they find themselves in LOF homozygotes that lack the *Rescue*/Cargo chromosome (thereby bringing about further drive of *Rescue*/Cargo), regardless of the current levels of Cas9. S2G–S2I Fig provides an example that illustrates this point.

In contrast, versions of split^50cM^*ClvR* in which the *Rescue*/Cargo/gRNAs are located at the site of the essential gene being targeted for LOF allele creation (the “same site” TARE configuration of [18]), are not able to accumulate drive potential in the form of LOF alleles as Rescue/Cargo/gRNA spreads. This makes same site versions of split *ClvR* much weaker drivers (S4 Fig). As discussed above, drive mediated by a distant site split *ClvR* results in a population in which *Rescue*/Cargo/gRNAs are surrounded by LOF alleles, since the *Rescue* and essential gene loci are distinct and do not directly compete with each other for representation in an individual. In contrast, with same site versions of split *ClvR*, drive of *Rescue* into a population results, by necessity, in an inexorable loss of LOF alleles and thus drive potential, because all the relevant alleles––WT, LOF and Rescue––are at the same locus: A gain in frequency of one allele type requires a corresponding decrease in that of others, resulting in weaker drive over a number of conditions (S4 Fig). For these reasons our focus below is on distant site versions of split *ClvR*.

Finally, we note that when two independently segregating elements are introduced together into a WT population in double homozygotes (as in these examples), there is transient linkage disequilibrium between the two such that for the first few generations they are found together in individuals more often than would be expected based on their overall population frequencies. Linkage disequilibrium promotes drive of *Rescue*/Cargo/gRNAs with split^50cM^ *ClvR* because some chromosomes bearing Cas9/gRNAs or Cas9 are transiently protected from death in LOF homozygotes by virtue of an increased frequency of association with *Rescue*/Cargo, while the LOF allele creation that mediates drive continues. In contrast, linkage disequilibrium in the *Killer-Rescue*/Cargo system slows the initial rate of killing and provides no drive benefit, since the killing needed for drive only occurs when both components exist (the *Killer* has not been lost), and the *Killer* and *Rescue*/Cargo have segregated away from each other. As a result of these forces, more non-*Rescue*/Cargo chromosomes are killed per unit of *ClvR* driver chromosome than per unit of *Killer*. Altogether, these effects result in higher equilibrium frequencies of *Rescue*/Cargo and the Cas9 driver with split *ClvR* for any given release percentage of double homozygotes (Figs 2D, 2E, S2A and S2B).

### Split^50cM^ *ClvR* can drive *Rescue*/Cargo/gRNAs to high frequency in the presence of significant fitness costs

We now consider a more realistic scenario in which each transgene-bearing allele results in a 5% fitness cost to carriers, for a total cost of 20% in double homozygotes, for the same range of release percentages (10%, 20%, 30%, 40%, 60%) over 300 generations. In the case of *Killer-Rescue/*Cargo, the *Rescue/*Cargo frequency rises transiently, and then rapidly decays due to natural selection following loss of the *Killer* from the population (Fig 2G and 2H). Single locus *ClvR* with comparable fitness costs (10% cost for each combined Cas9/gRNA/Cargo/*Rescue* allele) spreads rapidly to genotype fixation for all shown introduction percentages (Fig 2I). Both versions of split *ClvR* show an intermediate drive behavior that is weak at low introduction percentages, but remarkably strong at higher ones. Thus, when split^50cM^ *ClvR* V2 is introduced at low frequency (10%, 20%), the frequency of *Rescue/*Cargo/gRNAs rises transiently to levels that are somewhat higher than those found with *Killer-Rescue*/Cargo (Fig 2J), and then drops as the frequency of the Cas9 driver drops (Fig 2K) and the LOF alleles that mediate drive are eliminated from the population through natural selection in homozygotes (Fig 2L). Version 1 behaves similarly (S2 Fig). However, when either split *ClvR* is introduced at higher percentages (illustrated for ≥30%), the *Rescue*/Cargo/gRNAs spreads rapidly to levels approaching transgene genotype fixation. The frequency of the Cas9 driver decays to zero by ~ generation 100 (Figs 2K and S2E), but *Rescue*/Cargo/gRNAs is maintained near transgene fixation for more than 300 generations by the many LOF alleles generated by Cas9/gRNA activity in earlier generations (Figs 2L and S2F), which continue to select in LOF homozygotes against individuals that lack the *Rescue*/Cargo chromosome.

While this last force is important for maintaining *Rescue*/Cargo/gRNA at high frequency in an isolated population, it is easily subverted through the addition of WT, illustrated for a scenario in which WT are released at a 50% release frequency into the above populations at generation 150 (dotted lines), a point at which the Cas9/gRNA or Cas9 driver chromosome has been completely eliminated. Following an initial drop, *Rescue*/Cargo/gRNAs undergoes a transient increase as non-*Rescue*/Cargo-bearing chromosomes are removed in LOF homozygotes. However, as the frequency of LOF alleles decreases in favor of WT versions of the essential gene this is followed by an inexorable loss of *Rescue*/Cargo/gRNAs through natural selection.

**Fig 3:**
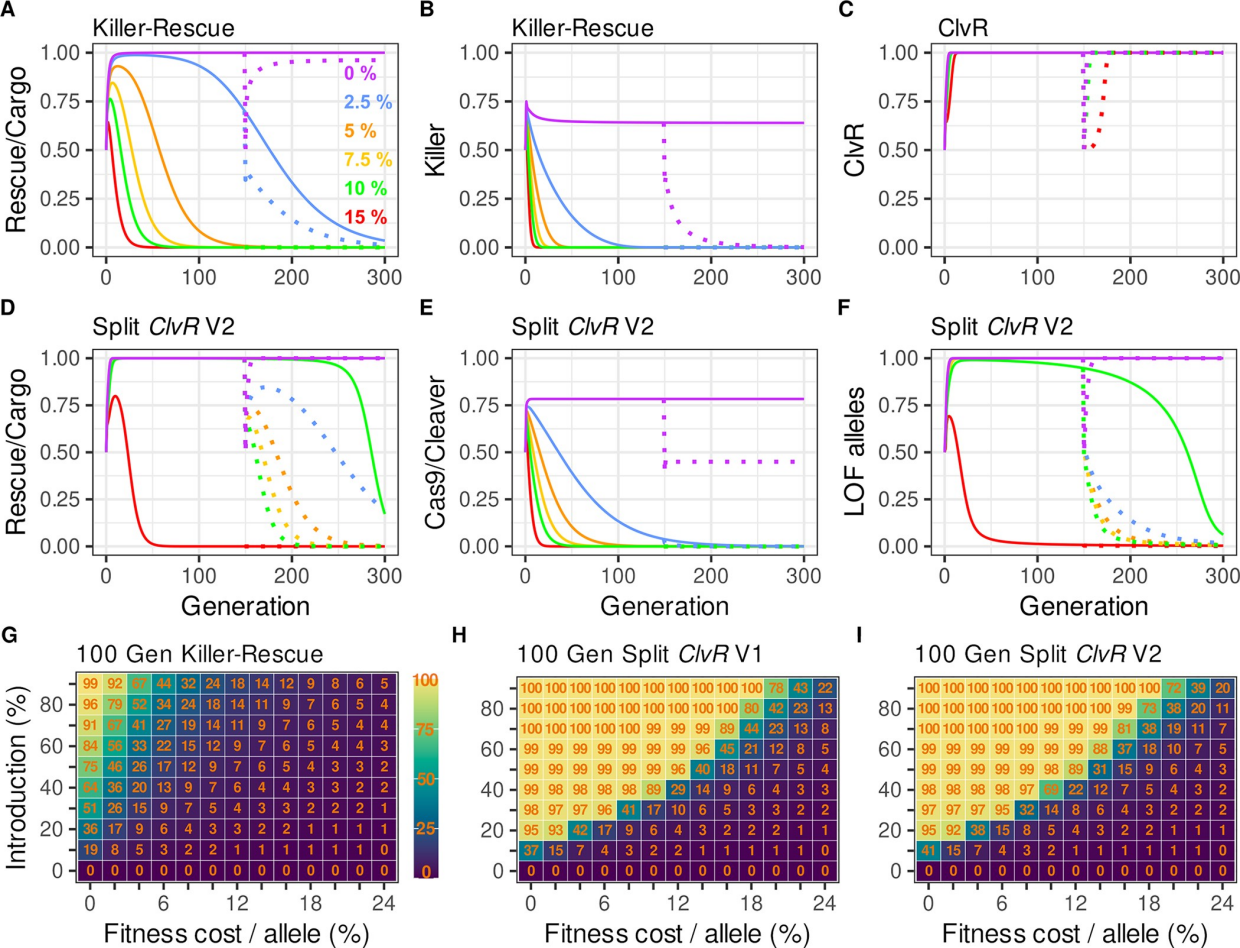
Population dynamics of *Killer-Rescue*/Cargo, ClvR and split^50cM^ *ClvR* for elements with different fitness costs, and introduction percentages. Split^50cM^ *ClvR* provides strong drive, with *Rescue*/Cargo/gRNAs spending prolonged time at high frequency even in the presence of significant fitness costs. **(A-F)** Population dynamics modeling of different drives introduced at a 50% release percentage, with fitness cost per allele varying from 0–15%. Fitness costs per transgene allele are 0% (purple), 2.5% (blue), 5% (orange), 7.5% (yellow), 10% (green), and 15% (red). Genotype frequencies of *Rescue*/Cargo and Killer or Cas9 are shown. Genotype frequencies are also shown for *ClvR*. Allele frequencies are shown for LOF. These are indicated with solid lines following the initial introduction. Genotype and allele frequencies following a 50% WT introductions at generation 150 are indicated in dotted lines. **(G-I)**. Heat maps showing the average *Rescue/*Cargo genotype frequency for the first 100 generations following releases of homozygotes for *Killer-Rescue*/Cargo **(G)**, split^50cM^ *ClvR* V1 **(H)**, and split^50cM^ *ClvR* V2 **(I)**, for different introductions and fitness costs/transgene allele. Each rectangle indicates the average Rescue/Cargo genotype frequency for the first 100 generations for the introduction and fitness cost associated with the tick marks. Thus, the box in the upper left designates a 90% introduction with a 0% fitness cost.

To explore split *ClvR*’s ability to drive in the presence of different fitness costs to carriers we now consider a scenario in which *Killer-Rescue*/Cargo, *ClvR* and split^50cM^ *ClvR* V2 are introduced at a constant release percentage of 50%, for a range of element-associated fitness costs (Figs 3 and S5 for split^50cM^ *ClvR* V1). The *Killer* in *Killer-Rescue*/Cargo is able to drive *Rescue*/Cargo to high frequency for ~80 generations when costs are absent or modest (2.5%/allele), but not when costs are larger (Fig 3A). These dynamics are reflected in the behavior of the *Killer*, which is rapidly lost except when fitness costs are absent or low (Fig 3B). *ClvR* spreads rapidly for all costs (Fig 3C). Split^50cM^ *ClvR* V2 spreads to near transgene fixation for >200 generations for all costs except for the highest, 15% per allele (Fig 3D). The basis for the strong drive by split^50cM^ *ClvR* V2 can be seen in the extended lifetime of Cas9 as compared with *Killer* (compare Fig 3B with Fig 3E), and the concurrent loss of almost all functional endogenous copies of the essential gene (Fig 3F). The key role LOF alleles play in maintaining *Rescue*/Cargo/gRNA at high frequency is well exemplified in the scenario involving a 10%/allele fitness cost (green line), in which the decrease in *Rescue*/Cargo/gRNA frequency (Fig 3D) (long after the Cas9 driver chromosome has been eliminated (Fig 3E)), is preceded by a decrease in the frequency of LOF alleles (Fig 3F). Finally, as also noted above (Fig 2J), loss of *Rescue*/Cargo/gRNA under conditions that would otherwise support long-term maintenance at high frequency can be hastened through the addition of WT at generation 150 (dynamics post 50% WT introduction in dotted lines in Fig 3D), a point at which the frequency of the Cas9 driver chromosome has been greatly reduced (2.5% cost/allele) or completely eliminated (costs/allele > 2.5%) (Fig 3E).

To get a more general sense of conditions able to support long-term maintenance of *Rescue*/Cargo/gRNA at high frequency we determined the average *Rescue*/Cargo/gRNA-bearing genotype frequency over the first 100 generations for different introduction percentages and fitness costs, an approach used to characterize several other drive mechanisms [37]. The results of this analysis, for a single release, are shown in Fig 3G–3I, for *Killer-Rescue* and split^50cM^ *ClvR* V1 and V2, respectively. In brief, *Killer-Rescue*/Cargo is able to maintain *Rescue*/Cargo at high frequency for a limited set of conditions that involve a high introduction percentage and low fitness costs (Fig 3G). In contrast, both versions of split *ClvR* drive *Rescue*/Cargo (Fig 3H) and *Rescue*/Cargo/gRNAs (Fig 3I) to sustained high transgene-bearing frequencies of ≥99% for a large range of introduction percentages and fitness costs.

To summarize, the results presented in Figs 2 and 3 show there are a broad range of conditions under which *Rescue*/Cargo/gRNAs of split^50cM^ *ClvR* spends considerable time at high frequency. That said, whenever the presence of *Rescue*/Cargo/gRNAs and Cas9 driver chromosomes results in some fitness cost to carriers, and LOF alleles have not spread to allele fixation (see the discussion for consideration of a stochastic case in which fixation has occurred) due to an earlier loss of the Cas9 driver from the population, LOF alleles and then *Rescue*/Cargo/gRNAs will ultimately be eliminated from the population through natural selection.

### The case for versions of split *ClvR* that result in drive with increased strength and duration

The introduction percentages discussed above represent a significant fraction of the population, though these levels are plausible in at least some cases, as they are substantially lower than those used in earlier nontransgenic insect population suppression programs [62]. Nonetheless, in contexts where the goal is to modify a population over a large, regional area (an extended area in which multiple target populations are connected by low or moderate levels of migration, but separated from non-target populations by little or no migration [see below for discussion of migration]), for a prolonged period, an ideal self-limiting drive system would have lower economic and logistical costs. By this we mean it would have greater strength at lower introduction percentages, and the drive element would persist and remain active for a longer period of time (requiring less supplementation over time to maintain efficacy). In short, drive strength and duration would behave more like that of self-sustaining *ClvR*, while remaining self-limited. Below we show how creation of linkage between Cas9 driver and *Rescue*/Cargo/gRNA components of split *ClvR* can achieve these goals in a manner in which drive strength and duration are tuned by the frequency of meiotic recombination between the components.

### Split versions of *ClvR* that include linkage (split^<50cM^ *ClvR*) have increased drive strength and duration

As with split^50cM^ *ClvR*, there are three possible configurations that involve linkage between split *ClvR* components. One of these, analogous to split^50cM^ *ClvR* V2 from Fig 1C, is shown in Fig 1E, and the others are shown in S1 Fig. In each configuration (denoted generally as split^<50cM^ *ClvR*), Cas9 driver and *Rescue*/Cargo/gRNAs components are located in cis, on the same chromosome, and the frequency of meiotic recombination between them is less than 50% (<50 cM). In the case of split^50cM^ *ClvR*, linkage disequilibrium (the difference between the frequency with which alleles of the two components are found together in an individual and that predicted by the product of their allele frequencies), following an initial introduction into a WT population, decays to zero very rapidly. However, when the frequency of recombination between the components is reduced, linkage disequilibrium decays more slowly. During the intervening generations (and as occurs every generation in the context of *ClvR*), linkage allows the Cas9 driver element to hitchhike with Cargo/*Rescue* to high frequency. The closer the loci are in cis (on the same chromosome), the more generations it takes for linkage equilibrium to be achieved. In the limit, drive strength and duration by split^<50cM^ *ClvR* approaches that of *ClvR* as the frequency of recombination approaches zero. However, so long as meiotic recombination between the loci occurs at some rate, linkage equilibrium is always reached, and the duration of drive is limited through the mechanisms discussed above, and below in the context of migration (see discussion of Fig 5).

**Fig 4:**
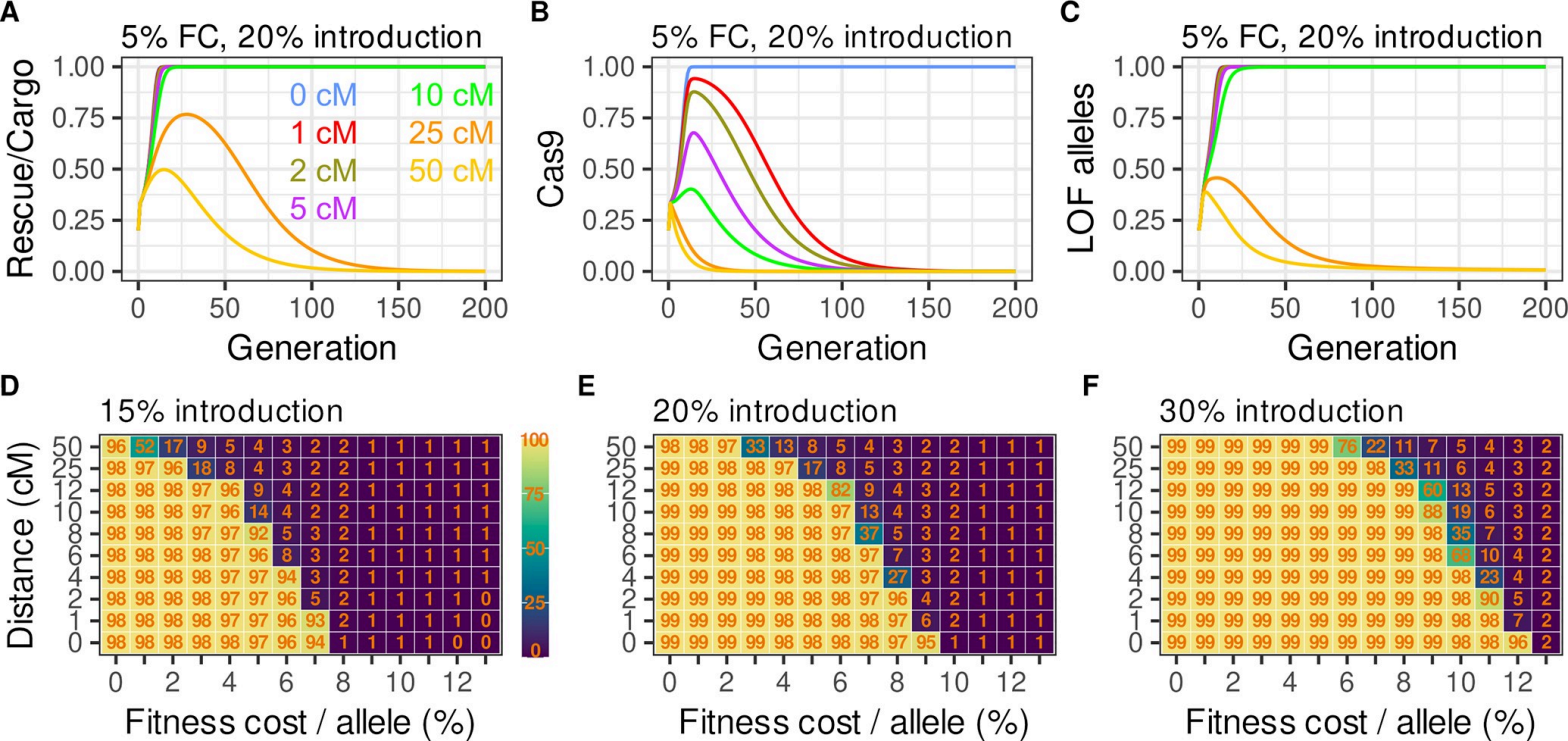
Behavior of split^<50cM^ *ClvR* V2 with linkage. Incorporating linkage into split *ClvR* (split^<50cM^ *ClvR* V2) results in a graded increase in drive strength, and duration of *Rescue*/Cargo/gRNAs at high frequency, as the degree of linkage increases (map distance decreases). **(A-C)** Versions of split^<50cM^ *ClvR* V2, in which the components are separated by different map distances, indicated by colored lines: 50cM (yellow), 25cM (orange), 10cM (green), 5cM (purple), 2cM (brown), 1cM (red) and complete linkage (blue). Each transgene allele has a fitness cost of 5%, and introductions are made at a release percentage of 20%. Y axis plots transgene-bearing genotype frequencies for *Rescue*/Cargo and Cas9, and allele frequencies for LOF. **(D-F)** Heat maps showing the average *Rescue*/Cargo/gRNAs genotype-bearing frequency over the first 300 generations for elements with different fitness costs and map distances between the components. Note that the y-axis not a linear scale. The distances shown are meant to capture a range of interesting biological values within a modest figure space. Release percentages occur at 15%, 20% or 30%.

These points are illustrated in Fig 4A–4C, for 7 different split^<50cM^ *ClvR*s, in which the map distances between the components range from zero (*ClvR*) to 50cM (split^50cM^ *ClvR* V2); each transgene allele results in a 5% additive fitness cost, and a single release is carried out at a (relatively low) 20% release percentage. Spread of *Rescue*/Cargo/gRNA to high frequency (>99% genotype frequency) fails when the map distance between the components is 50cM or 25cM, but occurs and is maintained for more than 600 generations (shown for 200 generations; see S7A–S7C Fig for 600 generation plots) when the map distance is 10cM or lower. The peak frequency reached and persistence time in the population both rise as the frequency of recombination between the Cas9 driver and *Rescue*/Cargo/gRNA decrease. The basis for increased drive with increased linkage can be seen in the plots of Cas9 genotype and LOF frequency (Fig 4B and 4C). When linkage is present Cas9 undergoes a transient increase in frequency as a result of hitchhiking with *Rescue/*Cargo/gRNA. This results in increased LOF allele creation, which creates stronger selection pressure favoring the *Rescue*/Cargo/gRNA-bearing chromosome.

A general sense of the relationship between map distance and fitness costs, and persistence time of *Rescue*/Cargo/gRNA at high frequency with split^<50cM^ *ClvR* V2 elements is shown in Fig 4D–4F, for three different introductions, in which we plot the average *Rescue*/Cargo/gRNA frequency for the first 300 generations (split^<50cM^ *ClvR* V1 in S6 Fig). To summarize, decreasing the frequency of recombination between the components allows lower frequency introductions to be used for elements with equivalent fitness costs, thereby decreasing costs associated with deployment. Decreasing the frequency of recombination also allows elements with higher fitness costs to spread and be maintained at high frequency for a given introduction percentage. This feature––the ability to provide extra drive strength for a given introduction percentage (the choice of which will be determined by economics and logistics)––is likely to be important in real world scenarios since fitness costs in the wild are probably often underestimated from laboratory experiments.

### Split *ClvR* and the challenges posed by bidirectional migration

Broadly speaking, there are two general contexts in which population modification with *ClvR* or split *ClvR* will be carried out. In the first, considered above, the target population encompases the entire range of the species, or multiple populations exist but target and non-target populations are separated by strong barriers to migration, such that the target population can be considered as a single population (because drive is negligible when *ClvR* is present at very low frequency [5,6,18,20]), spread to high frequency in the non-target population therefore being unlikely. In the second, considered below, the target population is linked to non-target populations by significant levels of migration. Here we focus specifically on bidirectional migration. Important questions in this context are how the influx of WT influences the ability of split *ClvR* to persist at high frequency in the target population, and what the consequences of drive in the target population are for transgene accumulation in non-target populations.

**Fig 5:**
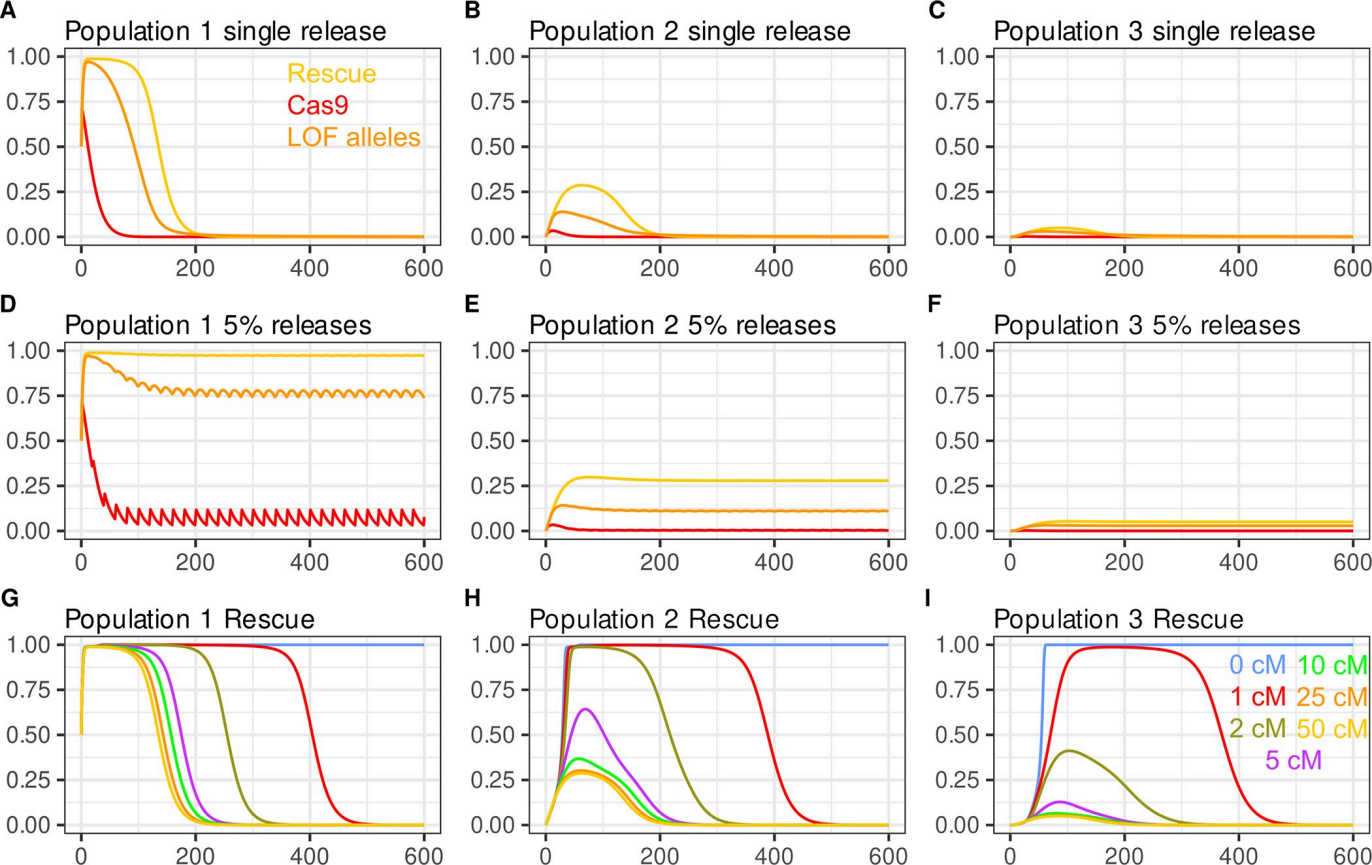
Dynamics of split *ClvR* in three populations connected by bidirectional migration. Increasing linkage with split *ClvR* results in increased *Rescue*/Cargo/gRNA lifetime at high frequency in the target population, but, at the migration rate shown (1%) also results in large amounts of spillover into neighboring populations. Shown are frequencies (y-axis) over generations (x-axis) in 3 populations connected by migration (1% migration rate between populations 1 and 2, and between 2 and 3) after an initial 50% release, for a split *ClvR* V2 with a 5% FC per allele. **(A-C)** Single release of split^50cM^ *ClvR* V2. *Rescue*-bearing genotypes (yellow), Cas9-bearing genotypes (red), and LOF alleles (orange). **(D-F)** Same as above but with additional 5% releases every 20 generations. **(G-O)** Single 50% release of split *ClvR*^<50cM^ with varying degrees of linkage: 50 cM (yellow), 25 cM (orange), 10 cM (green), 5 cM (purple), 2 cM (olive), 1 cM (red), 0 cM (blue). See S7 Fig for plots of Cas9 genotype and LOF allele frequencies.

To begin to explore these issues we follow, as an example, the behavior of split^50cM^ *ClvR* V2 in a 3 population model in which a single introduction into population 1 (the target population) is made at a release of 50%, with a fitness cost per allele of 5% (conditions used in some of the single population scenarios of Fig 3), and bidirectional migration occurs at a rate of 1% per generation between populations 1 and 2, and between 2 and 3 (with no direct link between 1 and 3). The frequency of *Rescue*/Cargo/gRNAs-bearing genotypes peaks at >99% and has a mean value of >90% for the first 100 generations, after which it rapidly decreases (Fig 5A). The lifetime of *Rescue*/Cargo/gRNAs and LOF alleles at high frequency in population 1 in a 3 population model is much shorter than in an isolated population (Fig 3D and 3I) due to the continuous back migration into population 1 of WT alleles at all three loci. At the same time, *Rescue*/Cargo/gRNAs, and to a lesser extent Cas9 driver and LOF alleles, are transferred through migration to population 2. Drive in population 2 due to the creation of new LOF alleles is negligible because the frequency of the Cas9 driver is low (Fig 5B). Levels of *Rescue*/Cargo/gRNAs, Cas9 driver chromosome, and LOF alleles are very low in population 3 (Fig 5C). These observations highlight a fundamental challenge for self-limiting drive mechanisms such as *Killer-Rescue*/Cargo, Split HEGs and split *ClvR*: how to keep the levels of *Rescue*/Cargo high in the target population in the face of continuous incoming migration once the initial input of driver chromosomes has been lost through natural selection and dilution into neighboring populations?

### Strategies to keep levels of split *ClvR Rescue*/Cargo/gRNAs high in the target population in the face of incoming migration

One solution to this problem is to maintain some frequency of the driver chromosome in the target population through repeated introductions. Fig 5D–5F shows an example in which, following the initial release of split^50cM^ *ClvR* (as in Fig 5A), further releases are carried out, every 20 generations, at a release percentage of 5%. These keep the frequency of *Rescue/*Cargo/gRNA-bearing genotypes ~98% indefinitely. They also lead to stable frequencies of *Rescue*/Cargo/gRNAs (26%), Cas9 driver (~1%) and LOF alleles (12.5%) in population 2, comparable to the peak frequencies observed following a single introduction (Fig 5A). Levels of *Rescue*/Cargo/gRNAs and LOF alleles remain very low in population 3, while those of the Cas9 driver chromosome are negligible.

A second solution is to incorporate linkage between *Rescue*/Cargo/gRNAs and the Cas9 driver chromosome. Hitchhiking of Cas9 with *Rescue*/Cargo drives Cas9 to higher frequencies in population 1. It also drives an increase in Cas9 frequency in neighboring populations, particularly when *Rescue*/Cargo/gRNAs and Cas9 are transferred while still linked in cis. Increased levels and persistence of Cas9 in target and non-target populations drive the continued creation of LOF alleles, which work to maintain *Rescue*/Cargo/gRNA at high frequency in both populations. These points are illustrated in Figs 5G–5I and S7, for a three population model in which versions of split^<50cM^ *ClvR* V2 having different recombination frequencies between the components are introduced into a WT population (population 1) at a release percentage of 50%, with each transgene allele resulting in a 5% fitness cost to carriers. For split *ClvR*s that incorporate some degree of linkage the *Rescue*/Cargo/gRNAs genotype (Fig 5G), Cas9 genotype (S7 Fig), and LOF allele frequency (S7 Fig) have prolonged lifetimes at high frequency in population 1 as compared with split^50cM^ *ClvR* V2 (Figs 5G and S7). Linkage also results in an increased frequency of *Rescue*/Cargo/gRNAs genotypes (Fig 5H and 5I), and Cas9 genotypes and LOF alleles (S7 Fig) in populations 2 and 3. However, in each case (except for that of *ClvR*; 100% linkage) drive is ultimately limited by the movement of WT alleles at the essential gene locus into the target population, which leads to the elimination of LOF alleles (the driver) through natural selection.

### The rate of migration influences drive outcome in target and non-target populations

**Fig 6:**
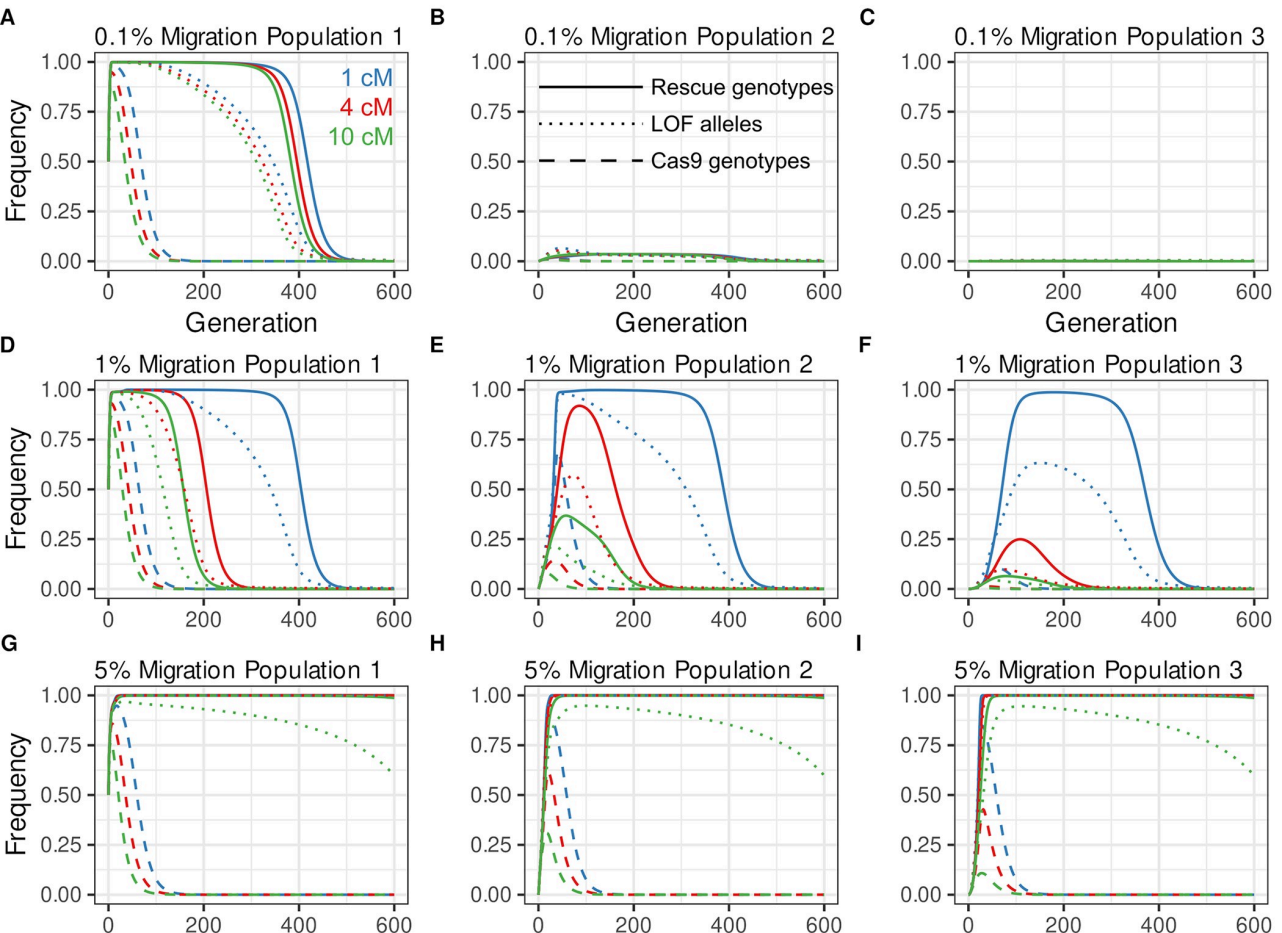
Drive dynamics for split^<50cM^ *ClvR*s with different genetic linkage and migration rates. The rate of migration can have a large effect on the time *Rescue*/Cargo/gRNA spends at high frequency in target and non-target populations. Migration rates are 0.1% **(A-C)**, 1% **(D-F)**, and 5% **(G-I)**. Linkage is 1cM in blue, 4cM in red, 10cM in green, *Rescue* genotypes are indicated with solid lines, Cas9 genotypes with dashed lines, and LOF alleles with dotted lines.

The importance of migration rate on drive outcome is illustrated in Fig 6 for representative split *ClvR* V2 elements with a 5% fitness cost per transgene, introduced at a constant percentage (50%) into population 1 of the 3 population model considered above. Bidirectional migration rates vary (0.1%, 1% and 5%), as does the recombination frequency between the components (1%, 4% and 10%). When the migration rate is very low (0.1% per generation), population 1 behaves as an isolated population, even for *ClvR* (Fig 6A–6C). The introduction threshold (due to the presence of element-associated fitness costs) needed for drive in populations 2 and 3 is never surpassed, and thus *Rescue*/Cargo/gRNAs fails to spread in these populations (Fig 6A–6C). With a 0.1% migration rate the recombination rate between the components has little effect on the persistence time of *Rescue*/Cargo/gRNAs at high frequency in population 1 (Fig 6A–6C) since this is ultimately determined by the rate at which LOF alleles are removed in favor of WT (brought in through migration from population 2) through natural selection, long after Cas9 has been eliminated.

In contrast, at (and above) some threshold migration rate, illustrated here for (5%) (see S8 Fig for a more extended version of this data in the form of heatmaps), the target and surrounding populations behave roughly as a single population, with sustained drive of *Rescue*/Cargo/gRNAs to high frequency occurring in all three populations (Fig 6G–6I). This occurs because the rate of movement of Cas9 and LOF alleles into populations 2 and 3 is very rapid, such that drive strength and duration are largely shared between the populations. This can be seen in the similar (though not identical) dynamics of Cas9 and LOF alleles in the three populations. Finally, when the migration rate is intermediate (shown here for 1%), the ability to maintain *Rescue*/Cargo/gRNAs at high frequency in population 1 is strongly dependent on the recombination rate between the components, with tight linkage (1cM) being required for long-term maintenance of *Rescue*/Cargo/gRNAs at high frequency (Fig 6D–6F). The basis for this can be seen in Fig 6E for the 4cM (red line) map distance. Drive in population 2 begins to wane around generation 100, due to reduced levels of Cas9 and LOF alleles (decreased drive strength) in population 1 and population 2. In consequence, *Rescue*/Cargo/gRNAs never achieves high levels in population 2. This, coupled with the high rates of back migration, which includes a large number of WT alleles at each locus, results in a rapid disappearance of LOF alleles which, in conjunction with element associated fitness costs, drive the frequency of *Rescue*/Cargo/gRNAs in population 1 down to near zero by generation 300. S8 Fig and its legend show and discuss an extended version of this data that covers additional map distances and migration rates.

Key conclusions from this data are that at very low migration rates (the specific values of which will depend on a number of variables including fitness cost and introduction frequencies) the target population behaves as an isolated population. At (and above) some threshold rate, the target and surrounding populations behave roughly as a single population (provided that drive is strong enough in a population of increased size; see S8 Fig). However, at intermediate migration rates, back migration of WT in the face of dwindling drive in population 1 can dominate, resulting in *Rescue*/Cargo/gRNAs having greatly reduced times at high frequency in population 1. This fate can be offset to some extent by reducing the recombination rate between the components, which increases drive strength and persistence in both population 1 and 2. Thus, while knowledge of the details of local migration rates may not be critical for bringing about initial drive of *Rescue*/Cargo/gRNAs to high frequency, it will be crucial to understanding its long-term fate, the scale and time frame of monitoring needed to ensure adequate ongoing coverage, and the possible need for further releases, either of transgenics to maintain drive, or of WT to limit drive in non-target populations.

### Chromosomal inversions that include the regions containing Cas9 and *Rescue*/Cargo/gRNAs from split^<50cM^ *ClvR* have minimal effect on drive behavior

The presence of a chromosomal inversion often leads to decreased rates of meiotic recombination within the region spanned by the inversion breakpoints, in inversion heterozygotes. In addition, when recombination within the inversion does happen, recombinant meiotic products (that involve a single or odd number of crossovers) do not contribute to viable progeny. In consequence genes located within an inversion are transmitted to viable progeny as though they were tightly linked (the parental haplotypes) (reviewed in [63]). In the context of split^<50cM^ *ClvR*, very rare events, necessarily happening in a single individual, may create chromosomes carrying an inversion whose breakpoints span the two linked split^<50cM^ *ClvR* components, and in which both split^<50cM^ *ClvR* components are present (a split^<50cM^ *ClvR* inversion haplotype). Such events would occur within an otherwise WT (non-inversion-bearing, for both target population and donor split^<50cM^ *ClvR*-bearing) chromosomal background. Conversely, wild populations are often polymorphic for (otherwise WT) chromosomes [63] that would be, with respect to the engineered split *ClvR*-bearing haplotype created in the lab, inversion-bearing. How does the presence of these chromosomal rearrangements affect drive?

### The fate of split^<50cM^ *ClvR* located within a paracentric inversion

We first consider the case in which an inversion arises that includes within its breakpoints the two components of a split^<50cM^ *ClvR*. Inversions can be pericentric, involving sequences on both sides of the centromere, or paracentric, involving only sequences from one chromosome arm. Here we focus on a “worst case” scenario, that of a paracentric inversion in an organisms such as *Drosophila*, in which meiotic recombination occurs in females but not in males. Pericentric inversions, which are often associated with a form of underdominance, are discussed in more detail in the legend of S9 Fig. In a paracentric inversion heterozygote, recombination within the inversion (assuming recombination is not completely blocked for reasons of chromosome mechanics) results in equal proportions of parental WT and inversion-bearing chromosomes, and an acentric and dicentric (which is ultimately resolved during meiosis II into deletion-bearing monocentric) chromosomes. In *Drosophila* and many other organisms, during female meiosis the planes of cell division are such that only one or the other of the parental haplotype chromosomes are pulled into the future oocyte, with the recombinant chromosomes being directed to the polar bodies [64]. In short, when meiotic recombination occurs within a paracentric inversion, in an inversion heterozygote, the outcome (one or the other parental chromosome haplotypes inherited by the oocyte) is functionally equivalent to that of a meiosis in which no recombination occurred within the inversion. Given this behavior (and the lack of meiotic recombination in males), a split^<50cM^ *ClvR* located within a paracentric inversion can most conservatively be considered as a self-sustaining *ClvR* element that spans the interval defined by the inversion. In this scenario the question then becomes what is the fate of this chromosome when it finds itself in a population of split *ClvR* (in a WT chromosome haplotype) that is being introduced into a target population (also consisting of a WT chromosome haplotype). In particular, does this new element spread to high frequency, making drive no longer self limiting?

**Fig 7:**
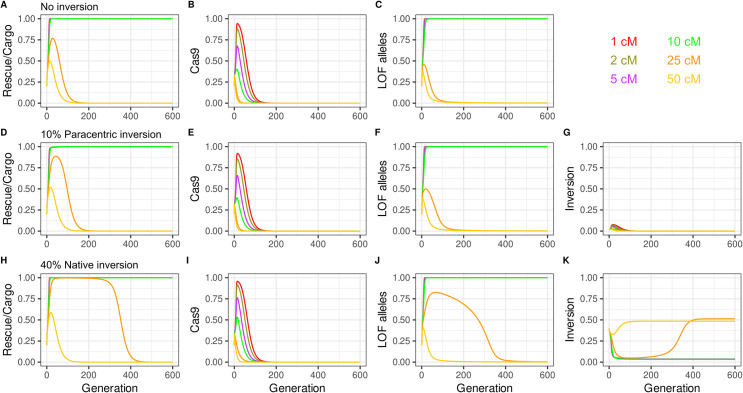
Split *ClvR* and Inversions. The presence of split *ClvR* within a paracentric inversion in the donor split *ClvR* population, or a non-split *ClvR* inversion in the target population, results in increased drive strength and duration that is still self-limited **(A-C).** *Rescue*/Cargo and Cas9 genotype frequencies **(A,B)** and LOF allele frequencies **(C)** for versions of split^<50cM^ *ClvR* with linkage, with each copy of *Rescue*/Cargo and Cas9 carrying a 5% fitness cost. Release percentages of 20% into a WT population **(D-G)**. Release of split<50cM as above, but with 10% of the split *ClvR*-bearing alleles being contained within a paracentric inversion. *Rescue*/Cargo and Cas9 genotype frequencies **(D,E)**, LOF allele frequencies **(F),** and inversion allele frequency **(G)** over time. **(H-K)** Introduction of split^<50cM^ *ClvR* as in **A-C**, but with the target population having a paracentric inversion that spans the region containing the split^<50cM^ *ClvR* present at a allele frequency of 40%.

Here we consider a “worst of the worst” case scenario, in which a split^<50cM^ *ClvR* arises in a single individual (as it always would), within a factory raising split^<50cM^ *ClvR* carriers. In this scenario, through some major mishap the population (which would begin as homozygous for the split^<50cM^ *ClvR* element on a WT chromosome haplotype) has undergone a severe population bottleneck, followed by expansion such that the split^<50cM^ *ClvR* inversion-bearing haplotype is now present at a frequency of 10% within the split^<50cM^ *ClvR* population. In this example (Fig 7), split^<50cM^ *ClvR* individuals are introduced into a WT population at a 20% release frequency (the total population therefore consisting at this point of 2% split^<50cM^ *ClvR* inversion-bearing haplotype). The split^<50cM^ *ClvR Rescue*/Cargo/gRNAs located on the WT haplotype spreads to high frequency for all recombination distances except 25cM and 50cM, as in the case where an inversion is not present (Compare Fig 7D–7G, with Fig 7A–7C). The split^<50cM^ *ClvR* located within an inversion also rises in frequency initially, since it also benefits from the removal of non *Rescue*-bearing chromosomes in LOF homozygotes (Fig 7G). In consequence, at these extraordinarily high split^<50cM^ *ClvR* inversion frequencies, the transient presence of the split^<50cM^ *ClvR* inversion haplotype contributes to drive strength and lifetime of *Rescue*/Cargo/gRNAs on a WT haplotype (compare Fig 7A with 7D for 25cM (orange) and 50cM (yellow). However, as the split^<50cM^ *ClvR Rescue*/Cargo/gRNAs on a WT haplotype achieves high frequency, that of the split^<50cM^ *ClvR*-bearing inversion haplotype undergoes a decline to very low levels. This happens because while the decay of linkage disequilibrium and loss of Cas9 through natural selection frees the *Rescue*/Cargo/gRNAs on a WT haplotype from fitness costs associated with Cas9, these costs are locked into the split^<50cM^ *ClvR* inversion-bearing haplotype. This haplotype gains only very rare drive benefit (shared with *Rescue*/Cargo/gRNAs on the other haplotype) from the presence of Cas9 once *Rescue*/Cargo/gRNAs and LOF alleles are ubiquitous. In consequence it undergoes a decrease in frequency through natural selection. This will happen so long as the costs associated with the split^<50cM^ *ClvR* inversion-bearing haplotype are greater than any benefit this haplotype receives due to the loss of non-*Rescue*/Cargo/gRNA haplotypes in LOF homozygotes (a benefit that would be always be shared with the lower fitness cost non-inversion *Rescue*/Cargo/gRNA haplotype). For all plausible scenarios (both components carry fitness costs), by the time the frequency of *Rescue*/Cargo/gRNAs on a WT haplotype begins to fade, the frequency of the inversion is below the threshold required for drive, resulting in its eventual loss. The code provided at Github allows the reader to explore many different fitness scenarios.

### Fate of a split^<50cM^ *ClvR* in a WT population polymorphic for an inversion

We now consider the converse situation, in which a target population carries an inversion (with respect to the split *ClvR* chromosome haplotype) at an allele frequency of 40% (with the rest being of the same chromosome configuration as the split *ClvR* haplotype). The presence of the inversion chromosome in the target population results in a transient enhancement of drive (Fig 7H–7J compare with Fig 7A–7C for 25cM (orange) and 50cM (yellow)). This happens because some inversion heterozygotes include versions of the split *ClvR* haplotype in which *Rescue*/Cargo/gRNA are still linked in cis. This results in a slowed (but not stopped) rate of decay of linkage disequilibrium (because in the case of a paracentric inversion recombinant gametes are not formed). This effect can be seen most clearly for the case of a 25cM split *ClvR*, in which *Rescue*/Cargo/gRNA and LOF alleles remain at high frequencies for much longer in the presence of the inversion as compared with its absence (compare Fig 7H–7J with Fig 7A–7C). However, whenever the *Rescue*/Cargo/gRNAs spreads to high frequency the WT (non-split *ClvR* bearing) inversion haplotype is driven down in frequency along with that of the WT (non-split *ClvR* bearing) non inversion haplotype. This occurs because from the point of view of the *Rescue*/Cargo/gRNAs locus all non carriers (inversion and non-inversion haplotypes that lack *Rescue/*Cargo/gRNAs) are competitors that the LOF alleles created at the essential gene locus work to eliminate. In short, while the presence of a WT inversion chromosome in the population transiently increases drive strength, the effect is self-limiting since when drive succeeds (Fig 7K, 1, 2, 5, and 10 cM) the frequency of the inversion is pushed down to very low levels that represent a balance between the cost associated with its occasional loss in LOF homozygotes that lack *Rescue*/Cargo/gRNAs (a fate the *Rescue*/Cargo/gRNAs haplotype never experiences) and the relative fitness benefit it gains due to the fact that it lacks fitness costs associated with the presence of *Rescue*/Cargo/gRNAs and/or Cas9. The dynamics associated with fitness costs and different haplotypes are also seen in the behavior of the inversion haplotype as *Rescue*/Cargo/gRNAs undergo their inevitable decrease in frequency due to natural selection. The inversion haplotype, which has a higher fitness because it lacks Cas9 or *Rescue*/Cargo/gRNAs, goes up in frequency to levels comparable to those of the pre-drive population. In summary, the presence of pre existing split *ClvR-*bearing or non-split *ClvR*-bearing inversion haplotypes in an otherwise non-inversion donor or recipient population, respectively, does not fundamentally prevent drive to high frequency, or prevent its eventual decay.

### Synthesis of split^50cM^ *ClvR* in *Drosophila*

To synthesize split^50cM^ *ClvR* V2 in *Drosophila* we used Cas9-mediated mutagenesis (see methods) to inactivate the Cas9 gene in flies carrying a single locus *ClvR* element on the 3rd chromosome (68E) that has the X-linked gene *tko* as its essential gene target (*ClvR*^*tko*^) [5]. Cas9 mutants in *ClvR*^*tko*^ were created by injecting into heterozygous *ClvR*^*tko*^*/+* embryos a Cas9-RNP-complex preloaded with two gRNAs targeting the Cas9 coding sequence. Heterozygous females carrying an intact *ClvR*^*tko*^ element give rise to >99% *ClvR*-bearing progeny [5]. Mutants in which Cas9 was mutated to LOF were therefore identified by outcrossing adult female progeny of injected *ClvR*^*tko*^*/+* embryos to WT males (*w*^*1118*^) and looking for Mendelian inheritance of the dominant marker carried within *ClvR*^*tko*^. Several such females were identified, and progeny from one, which also showed Mendelian transmission in outcrosses to WT, were used to generate a homozygous stock used for subsequent experiments, referred to as *Rescue*^*tko*^. The Cas9 open reading frame in these flies includes a 44 bp deletion at the target site of gRNA1, resulting in a premature STOP codon at amino acid 1339. This truncates the PAM-interacting domain [65] at the C-terminus of Cas9 by 30 amino acids (S10E Fig). The *Rescue*^*tko*^ insertion still carries gRNAs targeting endogenous *tko*, the recoded *Rescue*, and a cargo in the form of the dominant marker gene *OpIE-2-tomato*, as determined by sequencing.

The second component of the split *ClvR* system is located on the second chromosome, at 59D3 (attP docking line from [66], and carries a gene encoding Cas9 expressed under the control of germline-specific regulatory elements derived from the *nanos* gene (based on [67], modified as described in [68]), and a *3xP3-td-tomato* marker. This transgene and stocks that carry it are referred to as *Cleaver* (S10 Fig). Stocks homozygous for both components constitute the final split^50cM^ *ClvR* V2 and are referred to as *Cleaver;Rescue*^*tko*^ (see S11 Fig and Version 2 in Fig 1C with *Cleaver* on the 2nd chromosome and *Rescue*^*tko*^ on the 3rd).

### Genetic *behavior of Cleaver;Rescue*^*tko*^ components alone and in combination

**Fig 8:**
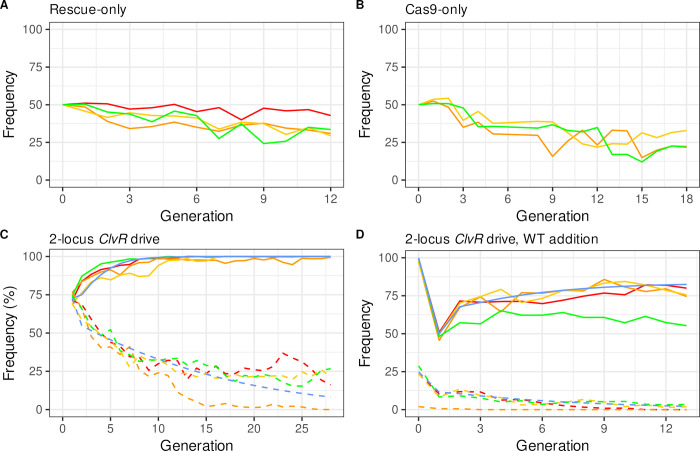
Population behavior of split drive components *Rescue*^*tko*^ and Cas9/*Cleaver* alone and together as a complete split^50cM^ *ClvR* V2 element. Split^50cM^ *ClvR Rescue*/Cargo/gRNAs spreads to very high frequency at the same time as Cas9 frequency undergoes a steep decline. Behavior of *Rescue*-only **(A)** and Cas9/Cleaver-only **(B)** behavior in a WT (*w*^*1118*^) background. **(C)** Genotype frequencies of *Rescue*^*tko*^ (solid lines) and Cas9/*Cleaver* (dashed lines) when introduced together as a complete **split**^**50cM**^ ***ClvR* V2 element,** in four replicates (red, green, orange, and yellow). Predicted drive behavior from a model in which *Rescue*^*tko*^ and *Cleaver* have additive fitness costs of 6.5% and 7.5%, respectively, are shown a blue lines (see methods for details). **(D)** Drive populations from **(C)** to which a 50% WT addition was made following generation 15.

As noted above, loss of Cas9 activity in *ClvR*^*tko*^, which creates *Rescue*^*tko*^, results in *Rescue*^*tko*^ being transmitted from heterozygous females to viable progeny in a Mendelian manner. To further demonstrate that this chromosome lacks drive activity we carried out a multi generation drive experiment. *Rescue*^*tko*^/+ males were mated with WT (*w*^*1118*^) females to bring about a *Rescue*^*tko*^ population allele frequency of 25% in the first generation. Four replicate populations were followed for 12 generations (Fig 8A). The population frequency of *Rescue*^*tko*^ underwent a consistent, modest decrease over time, similar to that of a control element used in our single locus *ClvR*^*tko*^ drive experiments [5,6], which carried the recoded *Rescue*, and a dominant marker, but not Cas9 or gRNAs. Similar drive experiments were performed with the Cas9-bearing *Cleaver* 2nd chromosome. Here, the population frequency of the transgene-bearing cassette also underwent a decrease over time, as expected for an element whose presence results in a modest fitness cost to carriers (Fig 8B). Finally, the signature genetic feature of a complete *ClvR* element is that when present in a heterozygous female, all surviving progeny should carry the *Rescue* element if cleavage-dependent LOF allele creation at the target locus is efficient in the female germline and in the zygote (non-carriers die because they lack a functional copy of the essential gene). Evidence that the levels of Cas9 expressed from the second chromosome *Cleaver*, along with gRNAs from the third chromosome *Rescue*^*tko*^ are sufficient to create LOF alleles at high frequency comes from results of experiments in which *Cleaver*/*+*; *Rescue*^*tko*^/+ heterozygote females were outcrossed with WT (*w*^*1118*^) males. As shown in S1 Table, all progeny were *Rescue*-bearing (n = 3093), for a cleavage and LOF allele creation rate of >99.97%.

### Drive performance of *Cleaver*; *Rescue*^*tko*^, a split^50cM^ *ClvR* V2

Results of the above modeling and experimental tests of *Cleaver;Rescue*^*tko*^ components predict that introduction of *Cleaver;Rescue*^*tko*^ into a WT population should result in drive of the *Rescue*^*tko*^ construct to high frequency. To test this hypothesis we mimicked an all male release by crossing double homozygous *Cleaver/Cleaver;Rescue*^*tko*^*/Rescue*^*tko*^ males to WT *w*^*1118*^ females. These mated females were then combined with mated WT females, and the mixed population was allowed to lay eggs into a bottle for one day and then removed. Progeny were allowed 13 days to develop to adulthood and mate. Adults were then anesthetized with CO2 and scored for the presence of *Cleaver* and *Rescue*^*tko*^ markers. They were then transferred to a fresh food bottle to repeat the cycle. Four replicate populations were followed. From all the scored genotypes we calculated the total number of flies carrying the *Rescue*-linked Cargo (*Rescue*^*tko*^ and *Cleaver;Rescue*^*tko*^; denoted as Cargo-bearing), and Cas9 (*Cleaver/+* and *Cleaver/Rescue*^*tko*^; denoted as Cas9-bearing). Results are plotted in Fig 8C. Starting from 69%-72% Cargo-bearing individuals in the first generation all four replicates reached >97% *Rescue*/Cargo-bearing by generation 11. At the same time the frequency of the *Cleaver* chromosome slowly decreased to between 30% and 0%. Three of the four replicates, in which Cas9 levels fell to between 26% and 18%, reached genotype fixation by generation 14. In contrast, in replicate B, in which the frequency of the *Cleaver* dropped almost to zero, the frequency of *Rescue*^*tko*^ remained high, but did not stabilize at genotype fixation. Population dynamics of individual replicates are presented in S11 Fig.

Successful population modification should also be reflected by an increase in *Rescue*^*tko*^ allele frequency and a decrease in that of *Cleaver*. We determined these frequencies at generation 25, after *Rescue*/Cargo genotype frequencies had been at or near transgene fixation for 10 generations. 100 males from each of the above drive populations were individually crossed to WT (*w*^*1118*^) females, and the offspring were scored for the presence of dominant markers (heterozygous males produce 1:1 transgenic:WT offspring; homozygous males produce 100% transgenic offspring). As shown in S2 Table, the frequency of the *Rescue*^*tko*^ allele increased dramatically, from between 34.6%-38.2%, to between 86.3% and 87.3% for the 3 populations in which *Cleaver* was still present at some level (allele frequencies between 9.3% and 17.5%), and to 83% for the population in which Cas9 was lost. These results are well explained by a model in which *Rescue*^*tko*^ results in a fitness cost to carriers of 6.5%, and *Cleaver* a fitness cost of 7.5%. Fitness costs were estimated by a non-linear least squares fit of our model to our data, (see Methods). That said, it is important to recognize that the estimated costs reflect behavior of these transgenes in a diversity of different genetic backgrounds with respect to Rescue/Cargo/gRNAs, Cas9 and LOF allele and genotype frequencies, which vary throughout the drive experiment. Thus, they constitute only a rough snapshot of fitness.

### Drive by the split *ClvR Cleaver*; *Rescue*^*tko*^ is transient

Together the above results demonstrate that a split^50cM^ *ClvR* V2 can be used to drive a *Rescue*/Cargo/gRNA transgene to high frequency, while the frequency of the Cas9 driver undergoes a contemporaneous decrease. How much remaining drive potential do these modified populations contain? To explore this question we took adults from the 4 drive populations above at generation 15 and combined them with an equal number of *w*^*1118*^ WT flies as the seed for the next generation of bottles. Offspring were then characterized as above for another 14 generations Fig 8D. As expected, addition of *w*^*1118*^ resulted in an immediate drop in the frequency of *Rescue*^*tko*^ and *Cleaver* individuals in the first generation. This is due to the presence of progeny from matings between *w*^*1118*^ individuals. In generation 2 the frequency of *Rescue*^*tko*^-bearing individuals increased. This also is expected and reflects the fact that many *Rescue*^*tko*^-bearing individuals in the seed generation were likely to be homozygous, with progeny from matings between them and *w*^*1118*^ now being heterozygous. The fate of *Rescue*^*tko*^ in the subsequent 12 generations depends on several forces. Fitness costs associated with *Rescue*^*tko*^ will drive its frequency down. At the same time, low levels of *Cleaver*, coupled with an initially high frequency of LOF alleles, will work to support a transient increase in *Rescue*^*tko*^ frequency. A transient rise in *Rescue*^*tko*^ frequency is hinted at in several of the replicates. However, what is not observed is a strong and consistent rise in *Rescue*^*tko*^ frequency from generations 3 onwards, as occurs in the presence of high frequencies of the *Cleaver* (Fig 8C), demonstrating that drive by split *ClvR* is transient.

## Discussion

Our results show that *ClvR* selfish genetic elements can be created that have different characteristics in terms of cost to initiate, maintain at high frequency, and ultimately eliminate a genetic modification in a target population. A complete *ClvR* element requires the smallest releases to bring about population modification for a given set of fitness costs, but is also self-sustaining, relatively invasive and thus challenging to eliminate. In contrast, all versions of split *ClvR*, including those with tight linkage, are self-limiting. In consequence, their ability to spread to high frequency in space is also limited. Modeling that takes into account features such as clines of chromosome structure polymorphisms such as inversions, density dependence, dispersal distance, spatial structure and possible context-dependence of associated fitness are required to more fully understand split *ClvR* behavior in specific ecological scenarios.

### Behavior of split *ClvR*, and split HEGs that cleave, home and rescue an essential gene, in finite and isolated populations

An important implication of the observations from Figs 2–4 on isolated populations––applied to more realistic, non-deterministic populations in which LOF allele fixation can be reached—is that if all WT alleles have been rendered LOF then every member of the population requires the presence of the *Rescue* in *Rescue*/Cargo/gRNAs for survival, a state of permanent transgene fixation that is independent of the presence of the Cas9 driver chromosome. The population will remain in this state even when fitness costs are present, and the *Rescue/*Cargo/gRNAs is not at allele fixation. Versions of split HEGs in which an essential gene is targeted for homing and *rescue* (c.f. [7,52–54]) should behave similarly if non-homed alleles are all rendered LOF through inaccurate repair following cleavage. This stands in contrast to the behavior of the *Rescue*/Cargo in a *Killer-Rescue*/Cargo system, in which, if a fitness cost-bearing *Rescue*/Cargo is present at anything less than allele fixation it will be lost through natural selection. The ability of LOF allele fixation to hold a split *ClvR* or split HEG targeting and rescuing an essential gene in a population at *Rescue*/Cargo transgene fixation—in an isolated population, independent of the presence of a driver chromosome, creates a unique and reversible system by which long-term study of the genetic and ecological impact of population modification can be explored. Following drive, *Rescue*/Cargo transgenes can be held at genotype fixation indefinitely, but they can also easily be eliminated by natural selection (provided their presence results in some fitness cost) following the addition of WT (see Figs 2 and 3). For these same reasons unplanned movement of low numbers of individuals to some other environment can never result in significant drive.

### What is the usable space of a self-limiting drive such as split *ClvR*?

Relevant considerations for real world applications include economics and logistics (costs), functional lifetime at high frequency (efficacy), and ability to spatially confine, modify and/or eliminate the modification from the population (control). Values for each of these variables will be given more or less weight depending on the specific social, regulatory, epidemiological and ecological context. Implementation costs for population modification by gene drive, using introduction frequency as a surrogate benchmark, span a large range of values (reviewed in [69]). Self-sustaining homing based drives in principle cost the least because they lack a threshold and spread rapidly from low frequency into all populations linked by even low levels of migration. Self-sustaining versions of *Medea/ClvR* type elements have intermediate costs; they typically will have an introduction threshold and are weak drivers at low frequency, though thresholds (and thus presumably costs) can be low if fitness costs are modest. High threshold underdominant self-sustaining systems, by definition require higher cost due to the large fraction of the population that must be transgenic (~30%-70%) in order for drive to occur, and the fact that introductions may need to be made into a number of areas, if they are connected by only low levels of migration, in order to guarantee complete coverage (the target may need to be “painted” with transgenics in order to achieve high frequency throughout) [41,69].

Split *ClvR* and the other self-limiting systems discussed (split HEGs, daisy drive, *Killer-Rescue*) span a middle range. Split HEGs and daisy drive require the lowest costs because drive is strong at low frequency. Versions of split *ClvR* (particularly in the presence of linkage) require higher costs than with split HEGs and daisy drive because drive of *ClvR*-like systems is weak at low frequency [5,6,18]. That said, the use of split HEGs (or self-sustaining HEGs) requires homing (which in some but not all versions also involves copying of the Cargo). In contrast, split *ClvR* does not involve homing, and Cargo is therefore replicated with high fidelity, as with other chromosomal DNA. These are features that will be important in species in which homing rates are low and/or loss of Cargo through incomplete homing results in decreased effective drive element lifetime. Finally, *Killer-Rescue* and some high threshold self-sustaining mechanisms that involve large releases involve yet higher costs; though the costs associated release of a high threshold self-sustaining drive mechanism can be amortized over time given its ability to persist indefinitely at high frequency. Costs for split *ClvR* can, in the presence of tight linkage, approach those of self-sustaining *ClvR* elements, but will probably always be more than those associated with mechanisms that involve high frequency homing.

The concept of costs is tightly linked to that of long term efficacy, and they will often track each other: reduced costs per unit of drive provide opportunities for increased coverage over time and space, which increases efficacy. That said, efficacy may in some contexts only be needed (or desired) for a short period of time; for example, in the context of testing. In such situations, weaker (and thus potentially more costly) self limiting drivers such (*Killer-Rescue*), or high threshold self-sustaining systems, may be of increased interest due to other characteristics related to control: weaker drive results (all other things, such as fitness cost, being equal) in more rapid loss once introductions have stopped. This, and/or a high threshold, also facilitate rapid reversibility, and strong(er) limits to spread in space as compared with other systems. In short, sometimes the ability to stop (or limit) drive and/or spread, and/or eliminate a modification may have a higher value than that of the costs for introduction and/or maintenance at high frequency, even for self-limiting systems.

With respect to control, in some contexts (malaria eradication), maximizing drive strength and duration at low cost may be the dominant variable. Self-sustaining homing based mechanisms (and perhaps other low threshold self-sustaining systems) are ideal because of their invasiveness and persistence. However, these same features make recall and/or stopping spread to high frequency within non-target populations (assuming such populations exist) challenging. A number of strategies have been proposed for altering the genetics of a population modified with a self-sustaining low threshold drive such as HEGs [42,70–77], Medea [1], and *ClvR* [6], but these do not generally return the population to the pre transgenic state, except (if at all) very indirectly. High threshold drive provides opportunities for real reversal through dilution with WT, but this requires human intervention.

Self-limiting drives of the type considered here represent a compromise. So long as some wildtype alleles remain––the drive element has not spread to fixation (overshot its mark) in the non-target as well as target populations (see the discussion above of fixation and loss in an isolated population for split *ClvR*, and Dhole et al for a similar discussion of Daisy drive)––they are guaranteed to fade (and in the unlikely case of inadvertent fixation removal can be initiated through direct addition of WT). However, the rate of loss is dependent on associated fitness costs, not the active underdominance that follows dilution with WT for high threshold self-sustaining systems. Also unlike with underdominant systems, active genetic mechanisms (killing of heterozygotes, their progeny or other specific genotypes) do not work to limit the consequences of bidirectional migration into and out of the target population. Instead, *Rescue*/Cargo alleles are free to float in neighboring populations at levels determined by migration rate and fitness, while the corresponding movement of WT into the target population serves to decrease the frequency of transgene-bearing genotypes, particularly when the frequency of the driver chromosome is low. The migration-mediated “passive diffusion” of drive components into neighboring populations contributes to the self-limiting nature of drive, by diluting the Cas9 and LOF alleles needed for drive as spread of *Rescue*/Cargo/gRNAs takes place. However, by its nature this same behavior (which also contributes to the self-limiting behavior of other split self-limiting drive methods such as *Killer-Rescue*/Cargo and Split HEGs) means that drive with these systems does not (unless migration rates are very low; see Fig 6) result in sharp borders with respect to gene flow into non-target areas. This is because the *Rescue*/Cargo component is free to persist—and in the case of split *ClvR* with tight linkage, and split HEGs and Daisy drive [37,43]—continue to drive for some number of generations in non-target populations. In some contexts this will be seen as a negative. But in others the ability of the *Rescue*/Cargo to spread in time and space, regardless of detailed knowledge about the presence or absence of specific physical, ecological barriers and dispersal distances (a challenge that may face high threshold underdominant systems in some contexts [41]), may be seen as a positive.

Thus, when considered in the context of control, self-limiting split drive mechanisms (including split *ClvR*) are probably best suited to several scenarios. One is environments in which migration rates in and out of the target region are low (with the conditions used herein, which are simply meant to serve as an example, these are ~ ≤0.1%), significant levels of transgenes outside the target area are not acceptable, and it is important that drive ultimately fade––unlike with self-sustaining low threshold *ClvR* or high threshold mechanisms––thereby guaranteeing an eventual end to drive potential. Or to put it another way, split *ClvR* is useful when there is a premium on inability to spread to *high frequency* in non-target areas in the face of possible––even if very unlikely––events that might result in breach of an otherwise strong migration barrier associated with containment of a self-sustaining system. A second context in which self-limiting drives such as split *ClvR* are likely to be useful are those in which the target region is ecologically complex (thereby supporting the use of low threshold drive mechanisms able to access all niches), large (thereby supporting the use of strong versions of self-limiting drives), and a significant frequency of transgenes in regions neighboring the target area is acceptable, but more global spread is not––thus the need for a self-limiting system to ensure drive will ultimately disappear in the absence of human intervention. This last feature in particular may sometimes be important as a kind of fail safe that differs in kind from the strategies associated with control of low and high threshold self-sustaining drive systems.

### Synthesis of self-limiting drive elements such as split *ClvR*

A further important consideration in choosing a drive mechanism for modification (and the inevitable next generation elements that will be needed to replace it as it loses efficacy) is ease of construction. Much progress has been made in the design of split HEGs. While drive into an otherwise WT population has not yet been demonstrated, results from the proof-of-principle experiments carried out thus far (in which Cas9 pre-exists in all members of the donor and recipient population) argue that by incorporating lessons learned (targeting essential sequences coupled with rescue, often with multiplexing of gRNAs) sustained population modification can be achieved [7,52–54]. Drive of *Killer-Rescue* into a WT population has also recently been shown [58]. For both these approaches, the components needed are straightforward to identify or create in diverse species, and can be made orthogonally acting and extensible, providing the possibility for multiple cycles of modification. Split *ClvR* also uses a very simple toolkit of three components to bring about drive: a site-specific DNA sequence modifying enzyme such as Cas9 and gRNAs that guide it to specific targets, sequences sufficient to bring about germline expression (which need not be germline specific) of Cas9 (maternal carryover is not necessary but enhances drive), and a recoded, cleavage resistant version of an essential gene able to rescue LOF phenotypes generated by DNA sequence modification of endogenous copies of the essential gene. The key components are orthogonally acting RNAs, essential genes, and *Rescues*, *and these are* each highly specific to the genes being targeted and rescued, and are indefinitely extensible (because any essential gene can serve as a target). Results in *Drosophila* show that self-sustaining *ClvR* elements (the components of which make up a split *ClvR* element, just in different locations) that drive to fixation can easily be generated [5,6,18], and can also bring about cycles of population modification, in which old content is replaced with new [6]. These features, in conjunction with our modeling showing that the strength and duration of drive can be tuned through incorporation of genetic linkage between the components, argue that split *ClvR* genetic elements represent a plausible platform for self-limiting gene drive in diverse species and regulatory regimes. Finally, while we have focused herein on describing how linkage can be used to create measured self-limited drive using the components that make up *ClvR*, we note that creation of linkage between the components that make up a split HEG––the Cas9 driver and the gRNA/Cargo that are being driven––can also be used to increase the strength and duration of self-limiting drive in this system, as will be presented in more detail elsewhere.

Split *ClvR* V2, in which Cas9 is located at one position and the gRNAs, Cargo, and *Rescue* at another, provides the most useful format for development, testing and implementation of split self limiting drive. Drive strength and duration are similar to those observed with other arrangements of components (V1 and V3), and the two strains required for drive can be kept separately as homozygous stocks that only show drive when brought together, providing a point of control. The V2 format also makes it straightforward to screen for components that work well together. The keys to success in building *ClvR* or split *ClvR* elements that drive are to have a high frequency of cleavage and LOF allele creation in *trans* by Cas9 and gRNAs, and efficient rescue of LOF in *cis* by the recoded *Rescue*. The ability of specific transgenes to bring about these activities can be influenced by nucleosome positioning [78], the local chromosome environment, and activity of specific gRNAs. With split *ClvR* V2 the activity of a number of *Rescue*/gRNAs and integration sites can be tested by crossing transgenics to individuals known from other experiments to have high levels of Cas9 expression in the germline. Transheterozygous females should give rise to all transgene bearing progeny in crosses to WT if there is a high frequency of cleavage and LOF allele creation in the maternal germline and early embryo, and 50% of the progeny should survive if the *Rescue* is efficient. Using a similar strategy, the ability of other DNA sequence modifying enzymes that do or do not use double strand breaks to bring about the creation of LOF alleles can be tested at sites where Cas9 is known to work well, following integration using site-specific recombinases or homologous recombination. Transgenes and genomic locations that support high levels of DNA sequence modifying activity can be similarly identified by analyzing the results of crosses between *Rescue*/Cargo/gRNAs at locations known to support efficient killing and rescue (as in this work, from our observations with 1-locus *ClvR*^*tko*^ [5,6,18]) and transgenics expressing the nucleases to be tested. Finally, when the goal is to create split *ClvR*s with linkage, components can be tested in *trans*, as above, before bringing them into linkage through recombination.

### Split *ClvR*, recombination and the influence of inversions

In order to take advantage of linkage to extend the strength and duration of drive with split *ClvR* the recombination rates between specific regions of the genome must be known. Rates across particular regions can be determined in several ways, depending on the tools available in the species [79–82]. They can vary significantly depending on genomic position, and are subject to variation by sex, environmental factors (reviewed in [83]), and in the presence of chromosome polymorphisms such as inversions [63]. Inversions are common in wild populations, and are a major force in evolution by virtue of their ability to lock otherwise unlinked or weakly linked traits into a shared haplotype [63]). Given this, it is important to understand how the presence of an inversion that spans the two elements of a split *ClvR* influences drive strength and life time. Similar concerns regarding inversions have been noted for the case of self-limiting systems for suppression, when X shredders coupled with Y-linked base editors are located within the X-Y pseudoautosomal region [59]. Interestingly, our modeling argues that while the presence of an inversion haplotype (either split *ClvR*-bearing or WT) can extend drive lifetime, the effects are relatively modest, and do not change the self-limiting nature of drive (Fig 7). That said, the topic warrants further study in more realistic populations that include spatial structure. Finally, we note that for any chromosomal drive element (*Medea*, *ClvR*, split *ClvR*, *Killer-Rescue*, underdominance, and some versions of homing in which the Cargo does not move with the HEG), the component being driven into the population will drag nearby chromosomal alleles from the donor genome along with it as it spreads, until recombination brings the drive element and these alleles into linkage equilibrium. Possible population effects of an increased frequency of donor chromosome alleles linked to the site of *Rescue*/Cargo insertion for the biology of the target population will need to be considered in any population modification strategy.

## Material and methods

### Generation of a *Cleaver* (Cas9) stock

This construct was derived from plasmid pnos-Cas9-nos ([67], Addgene #66208, a gift from Simon Bullock). We replaced the mini-*white* marker with *3xP3-td-tomato*, flanked the *nos* promoter und 3’UTR with gypsy insulators and added an attB site to facilitate integration into the fly genome. Details of the cloning procedure are described in [68]. The construct was injected in to a fly strain with an attP landing site on the 2nd Chromosome at 59D3 (Bloomington stock 9722, [66]) alongside a helper plasmid as phiC31 integrase source (Rainbow Transgenic Flies). Injected G0 flies were outcrossed to *w*^*1118*^ and progeny was screened for eye-specific expression of *td-tomato* to identify transformants. Transgenic F1 were balanced over *CyO* to get homozygotes.

### Generation of the *Rescue*^*tko*^ stock

The *Rescue* stock was based off of *ClvR*^*tko*^ described previously [5]. This stock has a complete *ClvR* selfish element, including Cas9, gRNAs, and the recoded *Rescue*. To implement split *ClvR* we decided to ablate Cas9 function from this stock, so that it would contain gRNAs and *Rescue* only. This was done by designing two gRNAs that target the Cas9 ORF (PAM in uppercase; gRNA1: tgattcatcagtcaattacgGGG, gRNA2: gtactgataaggctgacttgCGG) to create a LOF mutation in Cas9. CRISPR guide design [84] was done in the Benchling software suite. The two gRNAs were pre-mixed with Cas9 protein (all from IDT) to form RNP Cas9 complexes. Final concentration in the mixture was: Cas9 protein 500ng/ul, gRNA1 50ng/ul, gRNA2 50ng/ul. This mixture was injected into the offspring of homozygous *ClvR*^*tko*^ males crossed to *w*^*1118*^ females (Rainbow Transgenic Flies). To screen for potential loss of Cas9 activity in the injected offspring, we outcrossed the now *ClvR*^*tko*^/+ heterozygous G0 females to *w*^*1118*^ males. If the *ClvR* element and thus Cas9 is still functional we expect the progeny of this cross to be 100% *ClvR*-bearing due to germline and maternal carryover dependent killing of offspring that does not carry *ClvR*. If we see normal Mendelian inheritance where only 50% of offspring carry the *ClvR* marker, Cas9 function must have been lost. We recovered several G0 females that had lost *ClvR* activity. We chose one of them to build up a stock to carry out all the experiments in this study. Flies were balanced over TM3,*Sb* to get homozygotes. We also sequenced over the Cas9 ORF in this stock to map the mutation induced by the injection of the gRNA pair targeting Cas9 itself (S10 Fig). Construct sequence files, gRNA sequences, and alignments for this stock were published previously [5].

### Crosses to generate a double homozygous split *ClvR* stock

Homozygous *Rescue*^*tko*^*/Rescue*^*tko*^ and homozygous *Cleaver/Cleaver* flies were crossed to a double balancer *CyO*;TM3,*Sb*. Offspring with genotypes *CyO*;*Rescue*/TM3 and *Cleaver*/*CyO*;TM3 were crossed to each other to give double balanced *Cleaver/CyO*; *Rescue*/TM3 flies. These were crossed to each other to generate the double homozygous stock.

### Female germline cleavage rates

We crossed double homozygous *Cleaver/Cleaver;Rescue*^*tko*^*/Rescue*^*tko*^ males to *w*^*1118*^ females to get heterozygous females in the progeny. These heterozygotes were outcrossed to *w*^*1118*^ males and the progeny scored for the relevant dominant markers (see S2 Table and S1 Data for counts).

### Gene drive experiments

To start the gene drive experiment, we crossed a double homozygous *Cleaver/Cleaver;Rescue*^*tko*^*/Rescue*^*tko*^ stock to *w*^*1118*^ females. These mated females were mixed with WT *w*^*1118*^ mated females (mated with w1118 males) at a ratio of 2:1 and transferred to a fresh food bottle as the drive seed generation 0 for a starting allele frequency of 33%. These flies were allowed to lay eggs for one day and removed from the bottles. After 13 days the next generation of flies had eclosed. A random sample of flies (~300 on average) were scored for their genotypes on a CO_2_-pad and transferred to a fresh food bottle to continue the cycle. The actual population size was not determined but was about 2–3 times that of the transferred population.

After 14 generations flies were transferred to a fresh food bottle to continue the drive. However, instead of discarding them afterwards, we added an equal amount of *w*^*1118*^ and transferred them to another food bottle to seed the drive experiment from Fig 8B with 50% addition of WT. This drive experiment was performed as the one described above. All drive counts are in S1 Data.

### Computational model and data fitting

We wrote a discrete-generation, population frequency model for each of the drives examined in the paper in Python to predict their behavior under various conditions. We assumed random mating between individuals, equal mating access between all individuals within a population, cleavage of the target allele occurs during gametogenesis, fitness costs affect survival up to mating, and carryover of Cas9/gRNA activity occurs from females to zygotes but not from males to zygotes.

Our population dynamics model is a modified version of the series of difference equations previously used in our lab [60], which is itself a modification of the model designed by Deredec, Godfray, and Burt [85]. In an attempt to facilitate the encoding of the crosses of every male genotype against every female genotype while including every genetic modification enacted by a given gene drive, we developed a pair of three dimensional matrices to store all of the cross information of a given gene drive for each gene drive we were interested in studying. This approach is similar to that of MGDrivE [86], however our model is strictly deterministic, not species-specific, considers space differently (we assume large, 1–3 panmictic populations compared to MGDrivE’s more spatially explicit dynamics), we track haplotypes rather than just genotypes, and can adjust recombination distance between loci. This last feature allowed us to examine how recombination distance can affect the independent segregation of a driver allele vs a *Rescue*/Cargo allele as well as the consequences of inversions creating autonomous *ClvR* drives from split *ClvR*s.

Each matrix represents all possible male or female offspring, where the indices represent the father’s, mother’s, and offspring’s genotypes respectively. This means that index [0, 1, 0] of the male offspring matrix represents the male offspring of genotype ’0’ produced by the cross between a male of genotype ’0’ to a female of genotype ’1’; index [3, 0, 10] of the female offspring matrix represents the female offspring of genotype ’3’ produced by the cross between a male of genotype ’0’ to a female of genotype ’10’. These genotype numerical identities (and thus index in each matrix) are determined by their index in the list of male and female genotypes for each drive (these lists can be found in the ClvR_variables.py file). The actual entries in these indices are the sum of all possible offspring of the specified cross that are of the specified offspring genotype, which includes information about allele segregation and inheritance as well as gene drive activity. For example, consider the following two terms which come from index [0, 1, 0] of the male offspring matrix for the split *ClvR* autosomal v2 gene drive:

$$\frac{1}{2}\cdot \left(\frac{1}{2}\cdot f_{li}[0]\cdot f_{li}[1]\right)\cdot \left(\frac{1}{2}\cdot m_{li}[0]\cdot m_{li}[1]\right)+\ldots +\frac{1}{2}\cdot \left(\frac{1}{2}\cdot f_{un}[0]\cdot f_{li}[1]\right)\cdot \left(\frac{1}{2}\cdot m_{li}[0]\cdot m_{li}[1]\right)\cdot (1-f_{da}[0][1])\cdot f_{da}[1][1]+\ldots$$

For the first term, the first element $\frac{1}{2}$ identifies the percent of the offspring that are male, the second element $\left(\frac{1}{2}\cdot f_{li}[0]\cdot f_{li}[1]\right)$ identifies the proportion of offspring of a specific maternal haplotype, and the third element $\left(\frac{1}{2}\cdot m_{li}[0]\cdot m_{li}[1]\right)$ identifies the proportion of offspring of a specific paternal haplotype.

To clarify, *m*_*li*_ refers to the probability that a given pair of paternal alleles were inherited together from the same haplotype whereas *f*_*un*_ refers to the probability that a given pair of maternal alleles were inherited together from different haplotypes (here haplotypes refers to separate grandparental lineages). *f*_*li*_ and *m*_*li*_ range from 0.5 to 1, where 1 represents complete linkage of the two loci (hence the *li* subscript, corresponds to the loci being 0 cM away from each other) and the guaranteed co-inheritance of the alleles from a given haplotype, whereas 0.5 represents complete independence of the two loci (the loci being 50 cM away from each other) and the 50% chance that these alleles at these loci from a given haplotype will be co-inherited. Conversely, *f*_*un*_ and *m*_*un*_ range from 0.5 to 0, where 0 represents complete linkage of the two loci (corresponds to the loci being 0 cM away from each other), and thus the guarantee of never inheriting alleles from opposite haplotypes for these loci, whereas 0.5 represents complete independence of the two loci (the loci being 50cM away from each other) and a 50% chance that these alleles will be inherited from opposite haplotypes for these loci.

So for the term $\left(\frac{1}{2}\cdot m_{li}[0]\cdot m_{li}[1]\right)$, the $\frac{1}{2}$ represents the probability of picking a specific paternal allele from locus 1, *m*_*li*_[0] is the probability of picking an allele from locus 2 that is from the same haplotype as the allele picked from locus 1, and *m*_*li*_[1] is the probability of picking an allele from locus 3 that is from the same haplotype as the allele picked from locus 2. Similarly, for the term $\left(\frac{1}{2}\cdot f_{li}[0]\cdot f_{li}[1]\right)$, the $\frac{1}{2}$ represents the probability of picking a specific maternal allele from locus 1, *f*_*li*_[0] is the probability of picking an allele from locus 2 that is from the opposite haplotype as the allele picked from locus 1, and *f*_*li*_[1] is the probability of picking an allele from locus 3 that is from the same haplotype as the allele picked from locus 2.

The final complexity to this inheritance matrix are additional elements seen at the end of the second displayed term: (1−*f*_*da*_[0][1])·*f*_*da*_[1][1]. These two elements represent gene drive activities. *f*_*da*_ denotes female drive activity, where the first index determines which gene drive activity is being specified (the order of the gene drive activities for each observed drive is identified in the ClvR_general.ipynb file) and the second index identifies which female genotype is enacting the drive activity (while not used in our current figures, this allows different female genotypes to have different rates for the same drive activity). For this specific example, (1−*f*_*da*_[0][1])·*f*_*da*_[1][1] translates to the probability that the mother (of genotype ’1’) did not cleave the target allele during gametogenesis (the (1−*f*_*da*_[0][1]) element) but did cleave the target allele in the fertilized egg as a result of maternal carryover of cas9 and gRNAs (the *f*_*da*_[1][1] element).

To perform a simulation, we produce a generation specific frequency matrix, wherein a vector containing the frequencies of each male genotype is multiplied by a vector containing the frequencies of each female genotype. This matrix represents the frequency of each possible cross for the current generation and shares the same shape as the first two indices of the offspring matrices. Summing the in-place multiplication of these two tables iterated over each value for the third index of the offspring matrices yields the frequency of offspring of each genotype in the next generation (g1). These frequencies are then adjusted by their survival (as determined by the specified fitness costs) and normalized by the new total frequency to produce the frequency of adults of each genotype for g1, and these frequencies are then used to generate the next generation’s generation specific frequency matrix. For those simulations involving multiple populations, migration is enacted by removing a proportion of each genotype (based on the migration rate) and swapping them between populations, which occurs after the fitness cost is applied but before the next generation’s generation specific frequency matrix is calculated.

For our predictive modeling in the text, we varied fitness cost per allele, recombination distance between loci, and introduction frequency of different genotypes, while we used fixed germline cleavage (male and female) and carryover (female only) rates of 100%, based on our experimental observations.

For our fitness parameter estimation in Fig 8 we used the minimize function of the lmfit package in Python to do a least squares fit of our model to our genotype data. We estimated that our *Cleaver* and *Rescue*^*tko*^ alleles have ~7.5% (7.47% +/- 0.74%, 95% CI) and ~6.5% (6.56% +/- 0.038, 95% CI) fitness costs, respectively, relative to *w*^*1118*^ after performing a least-squares fit of our split *ClvR* model (using a data-averaged introduction frequency and assuming cleavage and carryover rates of 100%) to the Cas9-bearing and *Rescue*^*tko*^-bearing frequency data in the drive experiment (Fig 8).

### Imaging and figures

Images of fluorescent marker expression in whole flies (S10 Fig) were taken on a Leica M165FC with an AmScope MU1000 eyepiece camera and a DSRed filter. Composites were assembled in GIMP and rescaled to reduce file size. No additional image processing was performed. Modeling and drive figures were plotted in R with the “ggplot2” package. Color palettes for the heatmaps were from the “viridis” package.

### Fly crosses and husbandry

Fly husbandry and crosses were performed under standard conditions at 26°C. Rainbow Transgenic Flies (Camarillo, CA) carried out all of the embryonic injections for germline transformation. Containment and handling procedures for split *ClvR* flies were as described previously [68], with G.O and B.A.H. performing all fly handling.

## Supporting information

S1 Fig Split *ClvR*^<50cM^ configurations. Shown are the possible versions of split *ClvR* with elements on the same chromosome. See Fig 1 for split *ClvR* configurations with elements on different chromosomes.
(TIFF)

S2 Fig Population dynamics modeling of split^50cM^ *ClvR* V1 drive introduced at different release percentages, and an example illustrating the role that LOF alleles play in drive, independent of the Cas9 driver chromosome. **(A-F)** Plotted are genotype/allele frequencies (y-axis) over generations (x-axis). Release percentages are 10% (yellow), 20% (blue), 30% (green), 40% (red), and 60% (orange). In all panels in which fitness costs are present **(D-F)** there is a 50% release of WT in generation 150. Allele and genotype frequencies after this point are indicated with dotted lines. (**G-I)** One way to appreciate the power of the latent drive force provided by LOF alleles that segregate independently of the *Rescue*/Cargo is to consider a split^50cM^ *ClvR* population in which a *Rescue*/Cargo/gRNAs with no fitness cost has spread to allele fixation, the Cas9 driver chromosome has been completely eliminated (as would happen during drive if the presence of Cas9 resulted in a fitness cost to carriers, discussed below), and all endogenous copies of the essential gene have been rendered LOF (generation 0 in **G-I**). A large number of individuals WT at each of these loci is now introduced into the modified population (release of 50%). Following this introduction the frequency of *Rescue*/Cargo/gRNA and LOF alleles immediately drops. The frequency of LOF alleles continues to decrease over time as natural selection removes them when they find themselves in homozygotes. However, this same force works (transiently) to bring about a substantial increase in the frequency of the *Rescue*/Cargo/gRNA alleles **(G)** and genotypes **(H, I)** since only individuals lacking the *Rescue*/Cargo/gRNA-bearing chromosome are eliminated in the homozygous LOF background.
(TIFF)

S3 Fig Comparison of *Killer-Rescue* and split *ClvR* genetics. Crosses illustrate how split ClvR brings about greater drive than Killer-Rescue for a given introduction frequency through the creation of LOF alleles that can mediate drive in genotypes (and thus generations) that do not contain the driver (Killer or Cas9) locus. **(A)** Shown is a cross between a heterozygous carrier of a *Killer-Rescue* to WT. Offspring inheriting only the *Killer* allele die, ⅓ of the remaining offspring carry the *Killer*, ⅔ carry the *Rescue*, ⅓ remains WT. **(B)** Shown is a cross between a female heterozygous for split *ClvR* to a WT male. Cas9 mutates the target gene to LOF in the female germline. The target allele coming from the WT male gets mutated in the zygote due to maternal carryover of Cas9/gRNA complexes. This results in half of the offspring dying because they don’t carry a copy of the *Rescue*. Of the remaining progeny 100% carry the *Rescue* and 50% carry the *Cleaver*. **(C)** When a split *ClvR* male mates with a WT female, all the progeny survive. The target gene that was mutated in the male germline remains in the offspring (black circle). **(D)** When an individual heterozygous for the target gene mates again with a *ClvR* male, some of the offspring will end up with 2 mutated copies of the target gene and die. Only individuals that carry the *Rescue* are protected. **(E)** When individuals with one copy of the target gene mate with each other, ¼ of the progeny will die. This results in WT alleles at the Rescue locus being lost from the population even if the *Cleaver* allele was already eliminated (action at a distance).
(TIFF)

S4 Fig Split *TARE*. Modeling shows that split *ClvR* in a same site configuration (*Rescue*/Cargo/gRNAs at the same site as the essential gene being targeted) shows weaker drive than does distant site split *ClvR* under conditions shown in Figs 2 and 3. **(A-C)** Split^50cM^ *TARE* (Split *ClvR* with *Rescue*/Cargo/gRNAs located at the same site as the essential gene) for different introduction percentages from 10 to 60% and a fitness cost of 5% per allele (compare to Fig 2). **(D-F)** Split^50cM^ *TARE* for different fitness costs from 0–15% per allele and an introduction of 50%. (compare to Fig 3)
(TIFF)

S5 Fig Population dynamics modeling of split^50cM^ *ClvR* V1 introduced at a 50% release, with fitness cost per allele varying from 0–15%. Fitness costs per transgene allele are 0% (purple), 2.5% (blue), 5% (orange), 7.5% (yellow), 10% (green), and 15% (red). The behavior of V1 is comparable to that of V2 described in the text.
(TIFF)

S6 Fig Behavior of split^<50cM^ *ClvR* V1 with linkage. **(A-C)** Heat maps showing the average *Rescue*/Cargo frequency over the first 300 generations with different fitness costs and map distances between the components, introduced at release percentages of 15%, 20%, and 30%. The Y axis is not a linear scale. The distances shown are meant to capture a range of interesting biological values within a modest figure space.
(TIFF)

S7 Fig Cas9 and LOF allele behavior in a 3 population model. See Fig 5 for *Rescue/*Cargo/gRNA behavior. Shown are frequencies (Y-axis) over generations (x-axis) in 3 populations connected by migration (1% migration rate between populations 1 and 2, and between 2 and 3) after an initial 50% release, for a split *ClvR* V2 with a 5% FC per allele. Single 50% release of split *ClvR*^<50cM^ with varying degrees of linkage: 50 cM (yellow), 25 cM (orange), 10 cM (green), 5 cM (purple), 2 cM (olive), 1 cM (red), 0 cM (blue). **(A-C)** Cas9 genotype frequencies in the three different populations. **(D-F)** LOF allele frequencies in the three different populations.
(TIFF)

S8 Fig Heatmaps with different genetic linkage and migration rate. Heatmaps showing the average *Rescue* frequency for 100 **(A-C)** and 300 generations **(D-F)**. Linkage ranging from 0–50 cM, migration rate from 0.1–20% per generation. Note that both axes cover a wide range of values. These represent a range of biologically interesting values, and do not conform to a linear scale. When the migration rates are very low (0.1%), the recombination rate between the components has little effect on the persistence time of *Rescue*/Cargo/gRNAs at high frequency in population 1 since this is ultimately determined by the rate at which LOF alleles are removed in favor of WT (brought in through migration from population 2) through natural selection, long after Cas9 has been eliminated (see Fig 6). In contrast, when the migration rate is somewhat higher (shown in these heatmaps for between 0.5% and 4%) the ability to maintain *Rescue*/Cargo/gRNAs at high frequency in population 1 is strongly dependent on the recombination rate between the components. This is seen most dramatically with the average values for *Rescue*/Cargo/gRNAs frequency at generation 300 (D). When the migration rate is 1%, tight linkage (1cM) is required for long-term maintenance of *Rescue*/Cargo/gRNAs at high frequency. As the degree of linkage decreases (e.g. 4cM and 10cM), so does the average frequency of *Rescue*/Cargo/gRNAs. More generally, the data from Fig 6A–6F show that within the range of migration rates shown (0.5%-4%), increased rates of migration must be counterbalanced by decreased recombination frequency (increased drive strength and duration) in order for *Rescue*/Cargo/gRNAs to be maintained at high frequency. Interestingly, when migration rates are ≥5%, and the recombination rates are ≤12cM, sustained drive of *Rescue*/Cargo/gRNAs to high frequency occurs in all three populations: they behave as one large population. Finally, it is important to note that these plots are only meant to provide an example of how migration rate effects drive behavior. Drive behavior will be follow the same trends, but the details depend importantly on specific initial conditions.
(TIFF)

S9 Fig Pericentric inversions. Recombination within a pericentric inversion, in an inversion heterozygote, creates equal proportions of gametes that carry a WT or inversion chromosome (the parental haplotypes) or recombinant chromosomes that carry duplications and deletions (recombinant haplotypes). Both parental and recombinant haplotypes have a single centromere and are inherited by progeny with equal frequency; those that inherit recombinant chromosomes are typically unfit or dead due to genic imbalance. The zygotic loss of recombinant chromosome-bearing progeny results in *apparent* tight linkage between genes in the inversion since only progeny with parental haplotypes (WT or inversion) survive. Since the inversion-bearing chromosome is by definition rare (it arose spontaneously in a WT background), it suffers from a form of underdominance (it experiences a 50% loss frequency whenever recombination occurs in an inversion heterozygote), and is (all other things being equal) eliminated from the population. Based on this behavior underdominant pericentric inversion have in fact been explored as a form of high threshold gene drive for population modification [32,33]. S9 Fig illustrates these points for versions of split *ClvR* that find themselves within a pericentric inversion that spans different recombination distances, in populations of split^<50cM^ *ClvR* (WT chromosome haplotype) being introduced into a WT population (also WT chromosome haplotype). In each case, through a remarkable mishap, the split^<50cM^ *ClvR* inversion haplotype allele frequency is, at the time of introduction, now 10% of the total split^<50cM^ ClvR population. Split^<50cM^ ClvR is introduced at a frequency of 20%, and each transgene carries a 5% fitness cost. **(A)** *Rescue*/Cargo/gRNAs and **(B)** Cas9 genotype frequencies, and LOF allele frequency **(C)** are indicated. Inversion genotype frequency is shown in **(D)**. Note that the frequency of the inversion increases transiently due to the creation of LOF alleles, which act to promote the spread of any chromosome that carries a *Rescue*/Cargo/gRNAs, but then ultimately declines to very low levels. The special case in which the presence of a pericentric inversion results in a complete block to recombination in an inversion heterozygote is equivalent to that of the paracentric inversion considered in Fig 7, in which no recombinant gametes contribute to offspring.
(TIFF)

S10 Fig Split *ClvR* constructs, markers and alignment of Cas9 mutation. **(A)** Schematic of split *ClvR* constructs. The Cleaver (Cas9) is on the 2nd chromosome, *Rescue*/Cargo/gRNAs are on the 3rd. **(B-D)** Marker expression in different genotypes. **(B)** *Cleaver;Rescue* fly expressing eye-specific (*3xP3*) and ubiquitous (*OpIE*) *td-tomato*, **(C)** *Cleaver*-only fly expressing eye-specific *td-tomato* **(D)** *Rescue*-only fly expressing ubiquitous *td-tomato*. **(E) Cas9 LOF mutation in original *ClvR***^***tko***^ **locus.** The sequence alignment shows the mutation induced.
(TIFF)

S11 Fig Drive outcomes with all the scored genotypes. Legend on top of panels with *Rescue*/Cargo-bearing in red, Cas9-bearing in orange, *Cleaver/Rescue* in violet, *Cleaver*-only in green, *Rescue*-only in blue, and WT in yellow. **(A-D)** Replicates A-D. WT and *Cleaver;Rescue* in dotted lines for visibility.
(TIFF)

S1 Table Cleavage rates to LOF in females. Shown are the genotype frequencies in the offspring of a cross between heterozygous *Cleaver/+;Rescue*^*tko*^*/+* virgins and *w*^*1118*^ males. All of the offspring carried the dominant td-tomato marker of *Rescue*^*tko*^.
(PDF)

S2 Table Allele frequencies in the drive populations at generation 25. We measured allele frequencies of the drive elements by outcrossing 100 males from the different drive replicates at generation 25 to *w*^*1118*^ virgins and scored the offspring for their respective markers. C = *Cleaver* (Cas9, *3xP3-td-tomato*), R = *Rescue*^*tko*^ (*Rescue*, opie-tomato), + = WT. Examples of how scoring was performed are in S1 Data (allele frequencies).
(PDF)

S1 Data Drive counts, drive counts after WT addition, control drive counts, cross assay to determine cleavage to LOF, and assay to determine allele frequencies in the gene drive experiment. (XLSX)
