# Supplementary figures and images for: Split versions of Cleave and Rescue selfish genetic elements for measured self limiting gene drive
Source: PLoS Genet. 2021 Feb 18;17(2):e1009385. doi: 10.1371/journal.pgen.1009385 (PMC7951863; doi:10.1371/journal.pgen.1009385)

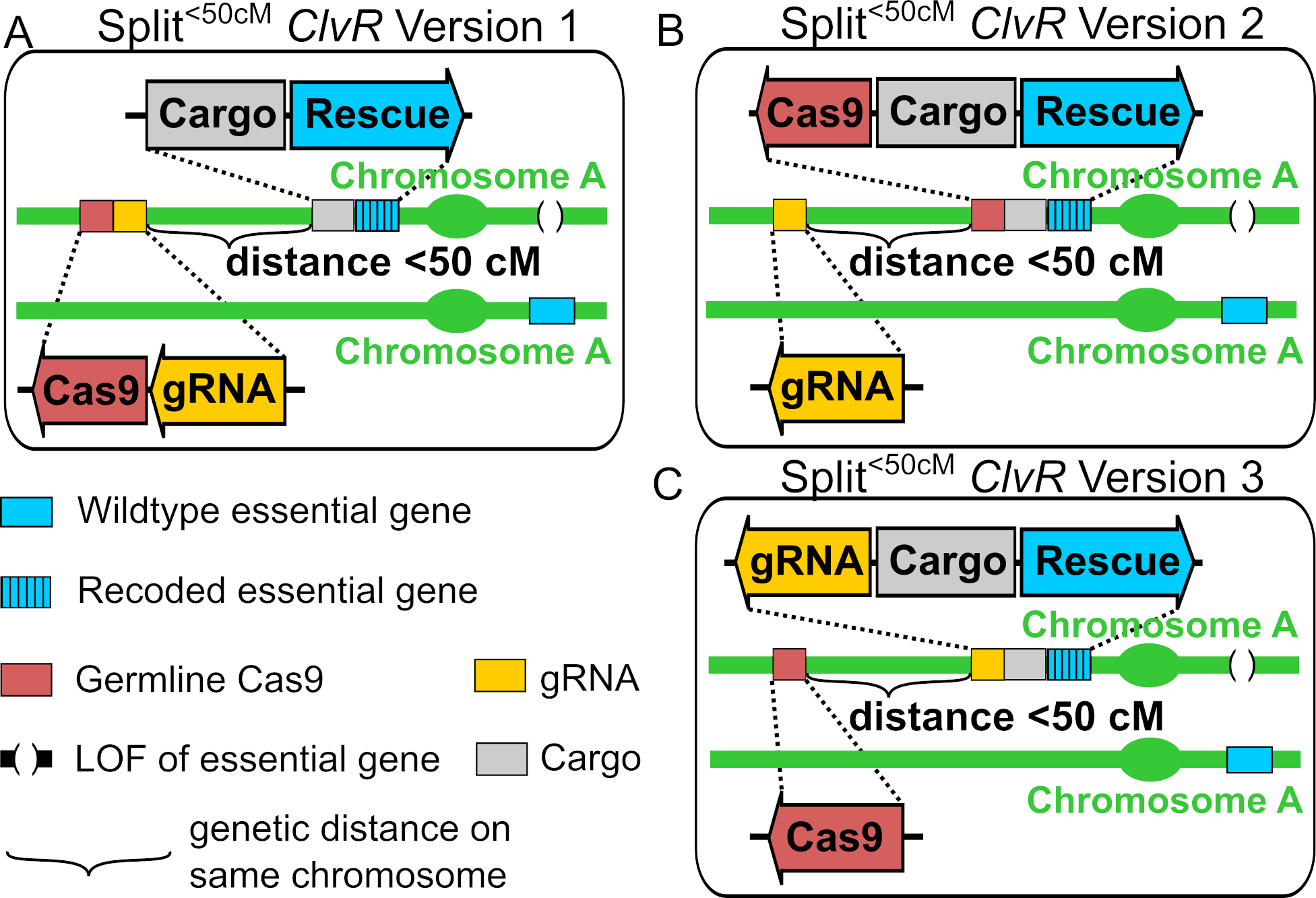

Supplement: S1 Fig — Shown are the possible versions of split ClvR with elements on the same chromosome. See Fig 1 for split ClvR configurations with elements on different chromosomes. (TIFF) [file pgen.1009385.s001.tiff]

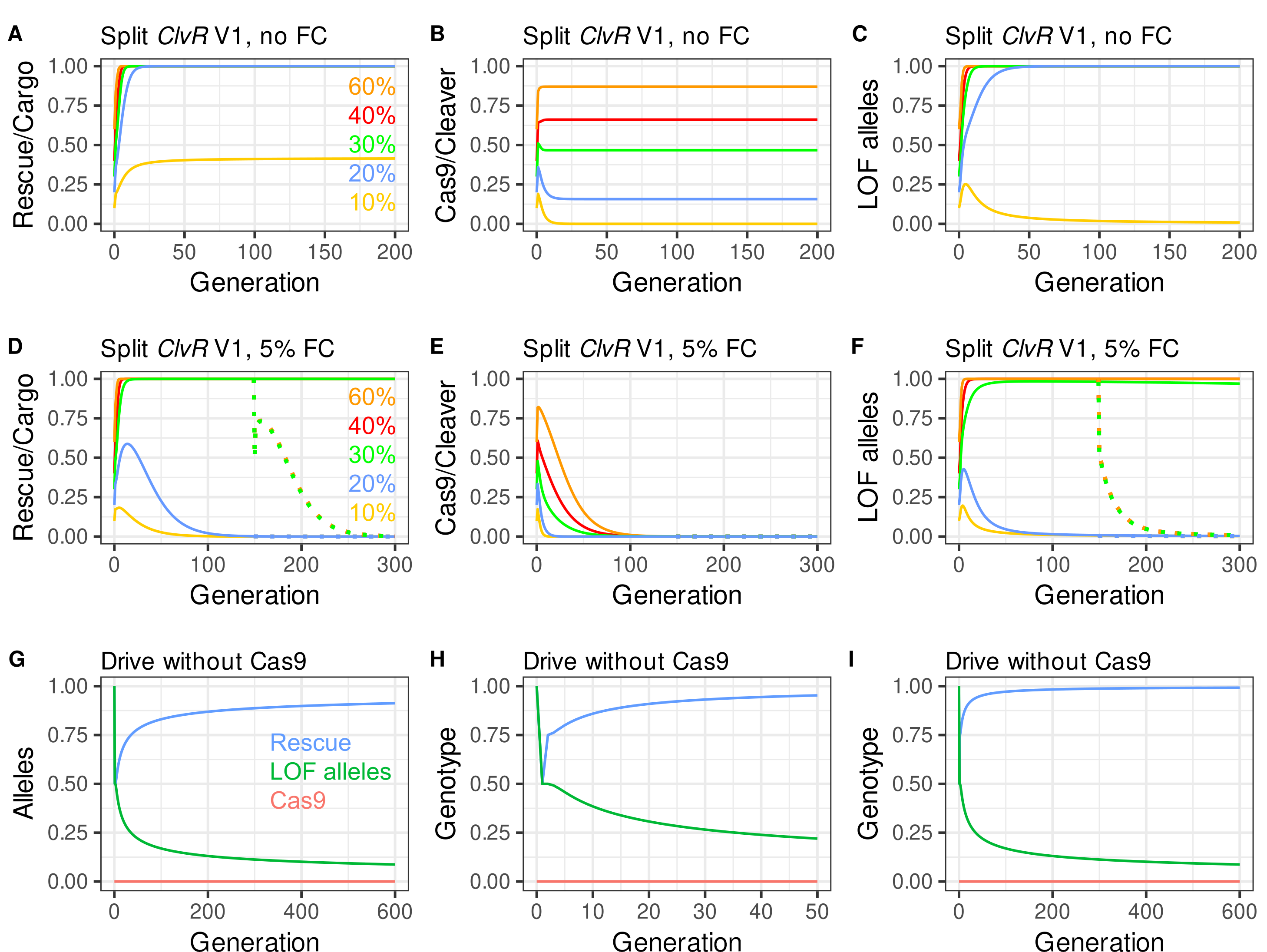

Supplement: S2 Fig — (A-F) Plotted are genotype/allele frequencies (y-axis) over generations (x-axis). Release percentages are 10% (yellow), 20% (blue), 30% (green), 40% (red), and 60% (orange). In all panels in which fitness costs are present (D-F) there is a 50% release of WT in generation 150. Allele and genotype frequencies after this point are indicated with dotted lines. (G-I) One way to appreciate the power of the latent drive force provided by LOF alleles that segregate independently of the Rescue/Cargo is to consider a split50cM ClvR population in which a Rescue/Cargo/gRNAs with no fitness cost has spread to allele fixation, the Cas9 driver chromosome has been completely eliminated (as would happen during drive if the presence of Cas9 resulted in a fitness cost to carriers, discussed below), and all endogenous copies of the essential gene have been rendered LOF (generation 0 in G-I). A large number of individuals WT at each of these loci is now introduced into the modified population (release of 50%). Following this introduction the frequency of Rescue/Cargo/gRNA and LOF alleles immediately drops. The frequency of LOF alleles continues to decrease over time as natural selection removes them when they find themselves in homozygotes. However, this same force works (transiently) to bring about a substantial increase in the frequency of the Rescue/Cargo/gRNA alleles (G) and genotypes (H, I) since only individuals lacking the Rescue/Cargo/gRNA-bearing chromosome are eliminated in the homozygous LOF background. (TIFF) [file pgen.1009385.s002.tiff]

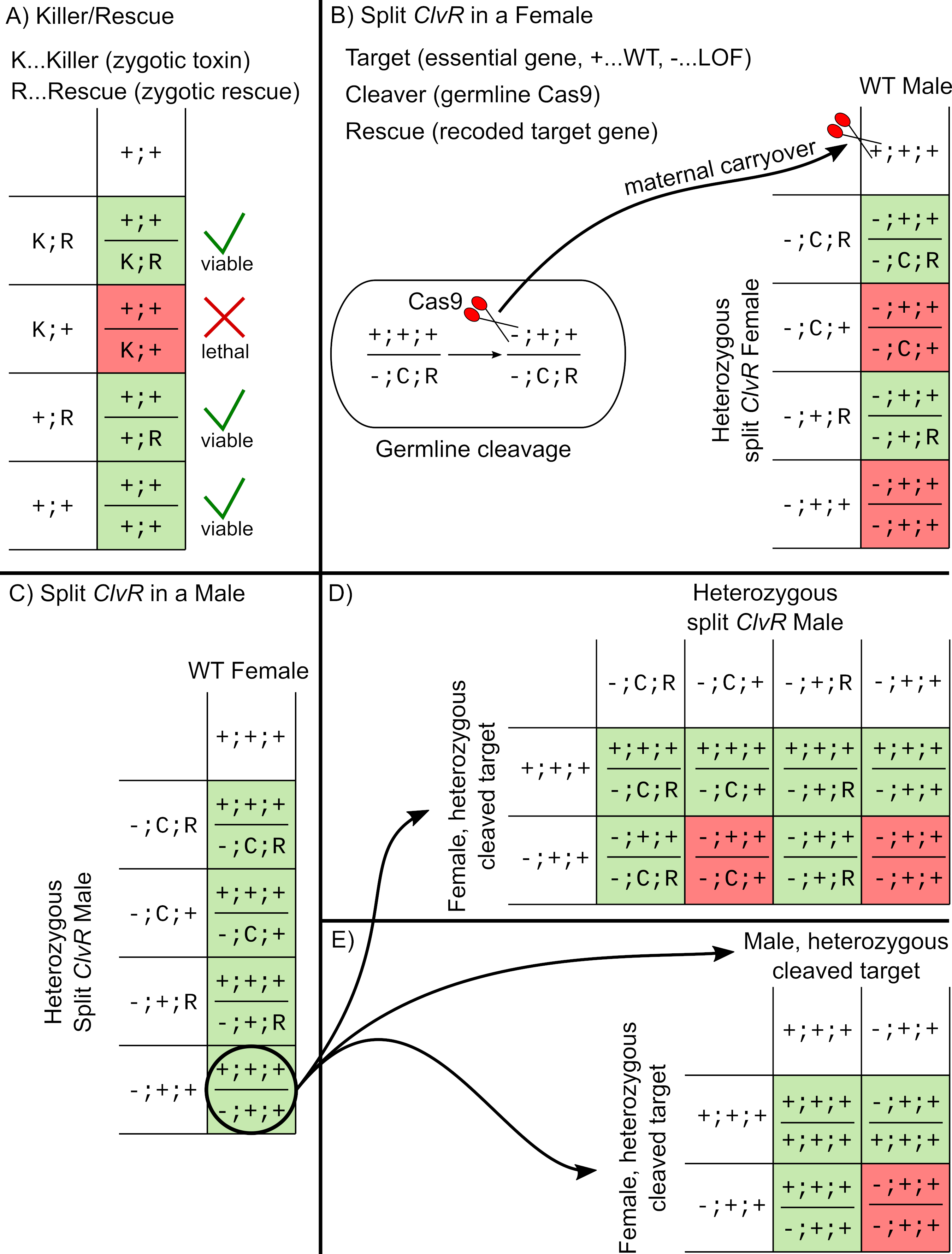

Supplement: S3 Fig — Crosses illustrate how split ClvR brings about greater drive than Killer-Rescue for a given introduction frequency through the creation of LOF alleles that can mediate drive in genotypes (and thus generations) that do not contain the driver (Killer or Cas9) locus. (A) Shown is a cross between a heterozygous carrier of a Killer-Rescue to WT. Offspring inheriting only the Killer allele die, ⅓ of the remaining offspring carry the Killer, ⅔ carry the Rescue, ⅓ remains WT. (B) Shown is a cross between a female heterozygous for split ClvR to a WT male. Cas9 mutates the target gene to LOF in the female germline. The target allele coming from the WT male gets mutated in the zygote due to maternal carryover of Cas9/gRNA complexes. This results in half of the offspring dying because they don’t carry a copy of the Rescue. Of the remaining progeny 100% carry the Rescue and 50% carry the Cleaver. (C) When a split ClvR male mates with a WT female, all the progeny survive. The target gene that was mutated in the male germline remains in the offspring (black circle). (D) When an individual heterozygous for the target gene mates again with a ClvR male, some of the offspring will end up with 2 mutated copies of the target gene and die. Only individuals that carry the Rescue are protected. (E) When individuals with one copy of the target gene mate with each other, ¼ of the progeny will die. This results in WT alleles at the Rescue locus being lost from the population even if the Cleaver allele was already eliminated (action at a distance). (TIFF) [file pgen.1009385.s003.tiff]

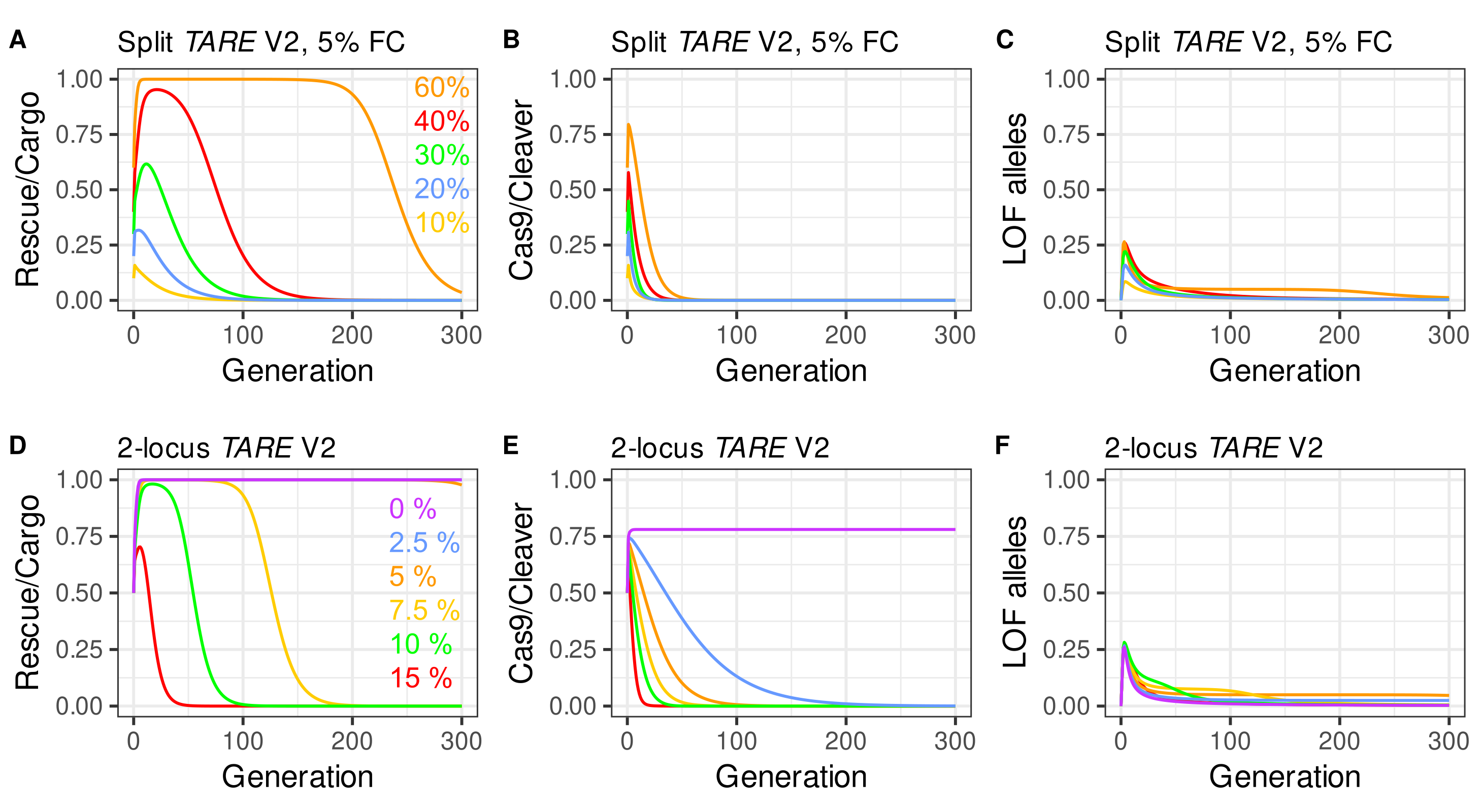

Supplement: S4 Fig — Modeling shows that split ClvR in a same site configuration (Rescue/Cargo/gRNAs at the same site as the essential gene being targeted) shows weaker drive than does distant site split ClvR under conditions shown in Figs 2 and 3. (A-C) Split50cM TARE (Split ClvR with Rescue/Cargo/gRNAs located at the same site as the essential gene) for different introduction percentages from 10 to 60% and a fitness cost of 5% per allele (compare to Fig 2). (D-F) Split50cM TARE for different fitness costs from 0–15% per allele and an introduction of 50%. (compare to Fig 3) (TIFF) [file pgen.1009385.s004.tiff]

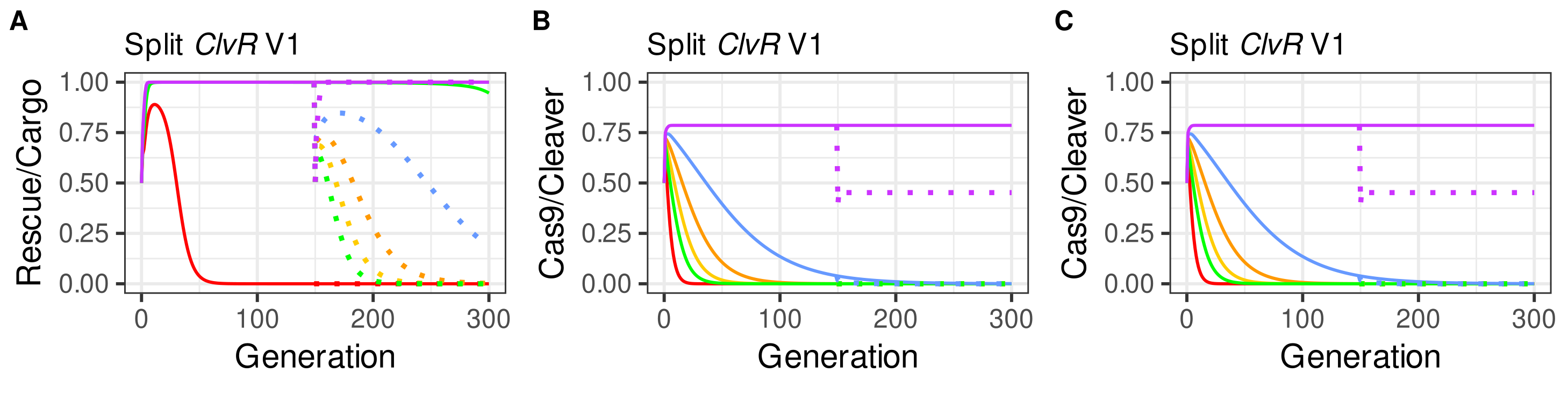

Supplement: S5 Fig — Fitness costs per transgene allele are 0% (purple), 2.5% (blue), 5% (orange), 7.5% (yellow), 10% (green), and 15% (red). The behavior of V1 is comparable to that of V2 described in the text. (TIFF) [file pgen.1009385.s005.tiff]

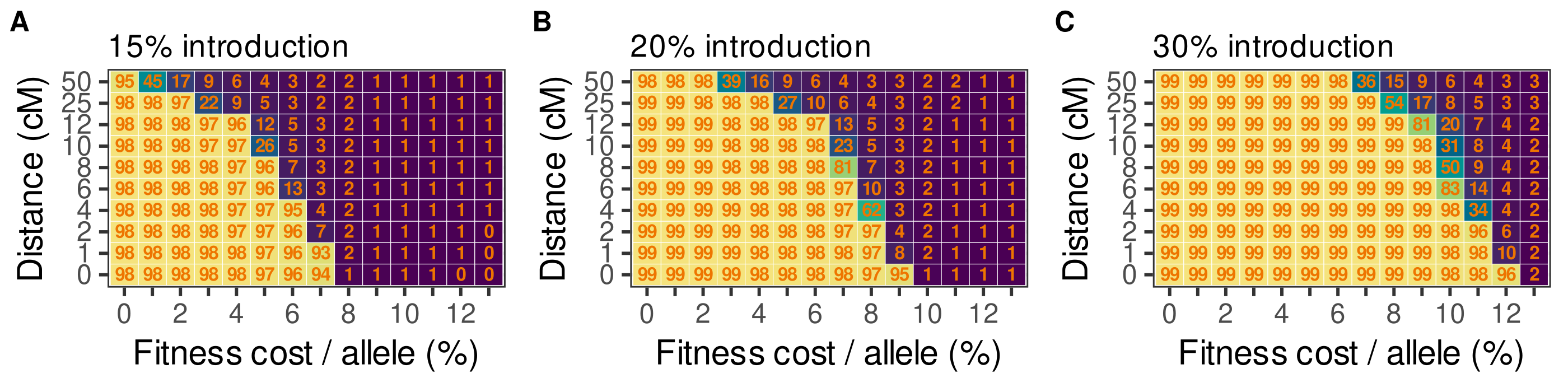

Supplement: S6 Fig — (A-C) Heat maps showing the average Rescue/Cargo frequency over the first 300 generations with different fitness costs and map distances between the components, introduced at release percentages of 15%, 20%, and 30%. The Y axis is not a linear scale. The distances shown are meant to capture a range of interesting biological values within a modest figure space. (TIFF) [file pgen.1009385.s006.tiff]

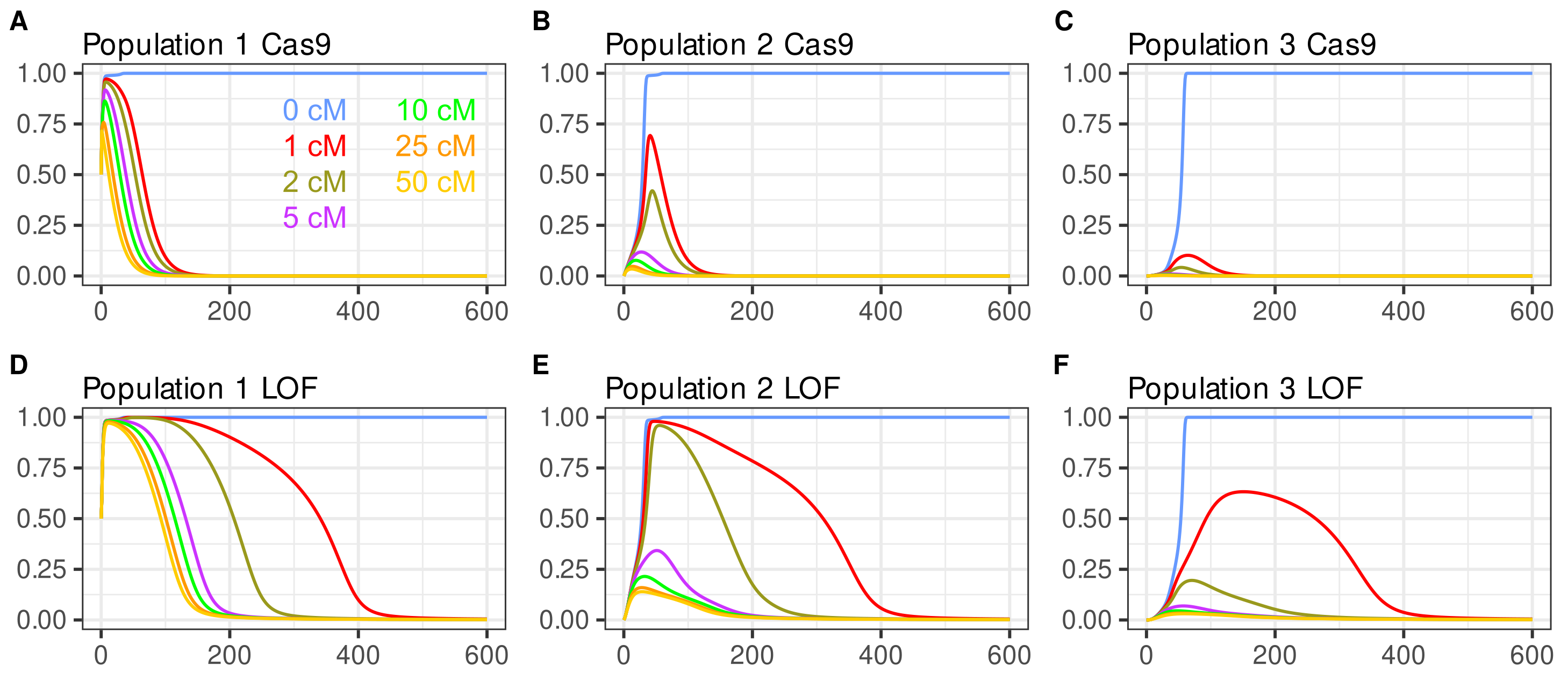

Supplement: S7 Fig — See Fig 5 for Rescue/Cargo/gRNA behavior. Shown are frequencies (Y-axis) over generations (x-axis) in 3 populations connected by migration (1% migration rate between populations 1 and 2, and between 2 and 3) after an initial 50% release, for a split ClvR V2 with a 5% FC per allele. Single 50% release of split ClvR<50cM with varying degrees of linkage: 50 cM (yellow), 25 cM (orange), 10 cM (green), 5 cM (purple), 2 cM (olive), 1 cM (red), 0 cM (blue). (A-C) Cas9 genotype frequencies in the three different populations. (D-F) LOF allele frequencies in the three different populations. (TIFF) [file pgen.1009385.s007.tiff]

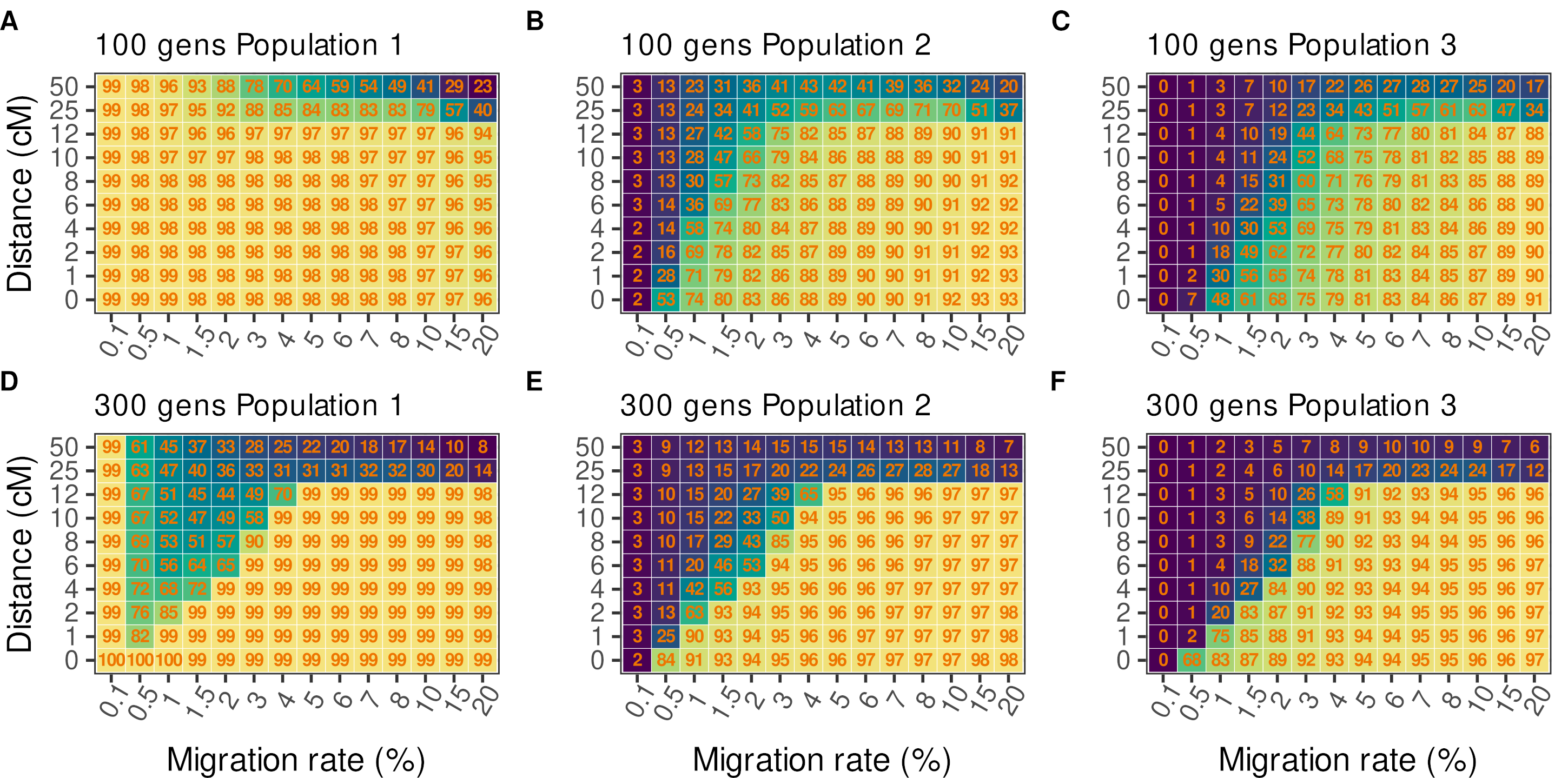

Supplement: S8 Fig — Heatmaps showing the average Rescue frequency for 100 (A-C) and 300 generations (D-F). Linkage ranging from 0–50 cM, migration rate from 0.1–20% per generation. Note that both axes cover a wide range of values. These represent a range of biologically interesting values, and do not conform to a linear scale. When the migration rates are very low (0.1%), the recombination rate between the components has little effect on the persistence time of Rescue/Cargo/gRNAs at high frequency in population 1 since this is ultimately determined by the rate at which LOF alleles are removed in favor of WT (brought in through migration from population 2) through natural selection, long after Cas9 has been eliminated (see Fig 6). In contrast, when the migration rate is somewhat higher (shown in these heatmaps for between 0.5% and 4%) the ability to maintain Rescue/Cargo/gRNAs at high frequency in population 1 is strongly dependent on the recombination rate between the components. This is seen most dramatically with the average values for Rescue/Cargo/gRNAs frequency at generation 300 (D). When the migration rate is 1%, tight linkage (1cM) is required for long-term maintenance of Rescue/Cargo/gRNAs at high frequency. As the degree of linkage decreases (e.g. 4cM and 10cM), so does the average frequency of Rescue/Cargo/gRNAs. More generally, the data from Fig 6A–6F show that within the range of migration rates shown (0.5%-4%), increased rates of migration must be counterbalanced by decreased recombination frequency (increased drive strength and duration) in order for Rescue/Cargo/gRNAs to be maintained at high frequency. Interestingly, when migration rates are ≥5%, and the recombination rates are ≤12cM, sustained drive of Rescue/Cargo/gRNAs to high frequency occurs in all three populations: they behave as one large population. Finally, it is important to note that these plots are only meant to provide an example of how migration rate effects drive behavior. Drive behavior will be follow t [file pgen.1009385.s008.tiff]

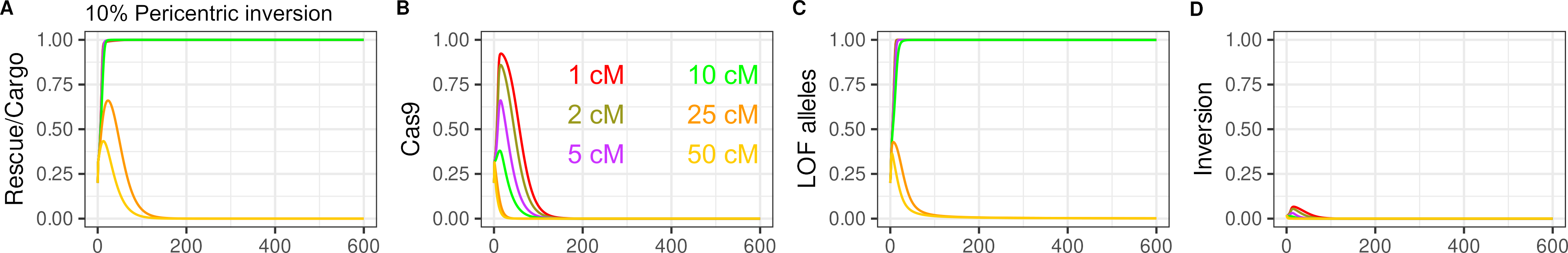

Supplement: S9 Fig — Recombination within a pericentric inversion, in an inversion heterozygote, creates equal proportions of gametes that carry a WT or inversion chromosome (the parental haplotypes) or recombinant chromosomes that carry duplications and deletions (recombinant haplotypes). Both parental and recombinant haplotypes have a single centromere and are inherited by progeny with equal frequency; those that inherit recombinant chromosomes are typically unfit or dead due to genic imbalance. The zygotic loss of recombinant chromosome-bearing progeny results in apparent tight linkage between genes in the inversion since only progeny with parental haplotypes (WT or inversion) survive. Since the inversion-bearing chromosome is by definition rare (it arose spontaneously in a WT background), it suffers from a form of underdominance (it experiences a 50% loss frequency whenever recombination occurs in an inversion heterozygote), and is (all other things being equal) eliminated from the population. Based on this behavior underdominant pericentric inversion have in fact been explored as a form of high threshold gene drive for population modification [32,33]. S9 Fig illustrates these points for versions of split ClvR that find themselves within a pericentric inversion that spans different recombination distances, in populations of split<50cM ClvR (WT chromosome haplotype) being introduced into a WT population (also WT chromosome haplotype). In each case, through a remarkable mishap, the split<50cM ClvR inversion haplotype allele frequency is, at the time of introduction, now 10% of the total split<50cM ClvR population. Split<50cM ClvR is introduced at a frequency of 20%, and each transgene carries a 5% fitness cost. (A) Rescue/Cargo/gRNAs and (B) Cas9 genotype frequencies, and LOF allele frequency (C) are indicated. Inversion genotype frequency is shown in (D). Note that the frequency of the inversion increases transiently due to the creation of LOF alleles, which act to promote the spread [file pgen.1009385.s009.tiff]

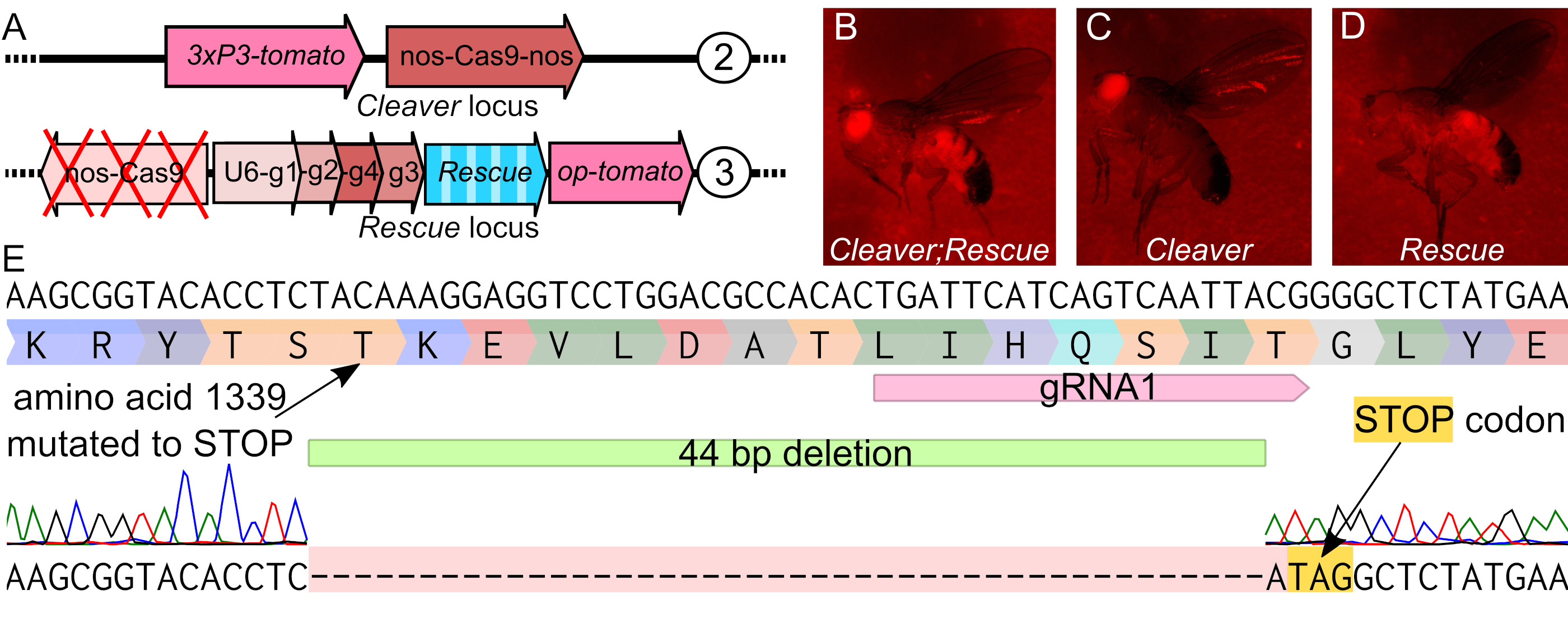

Supplement: S10 Fig — (A) Schematic of split ClvR constructs. The Cleaver (Cas9) is on the 2nd chromosome, Rescue/Cargo/gRNAs are on the 3rd. (B-D) Marker expression in different genotypes. (B) Cleaver;Rescue fly expressing eye-specific (3xP3) and ubiquitous (OpIE) td-tomato, (C) Cleaver-only fly expressing eye-specific td-tomato (D) Rescue-only fly expressing ubiquitous td-tomato. (E) Cas9 LOF mutation in original ClvRtko locus. The sequence alignment shows the mutation induced. (TIFF) [file pgen.1009385.s010.tiff]

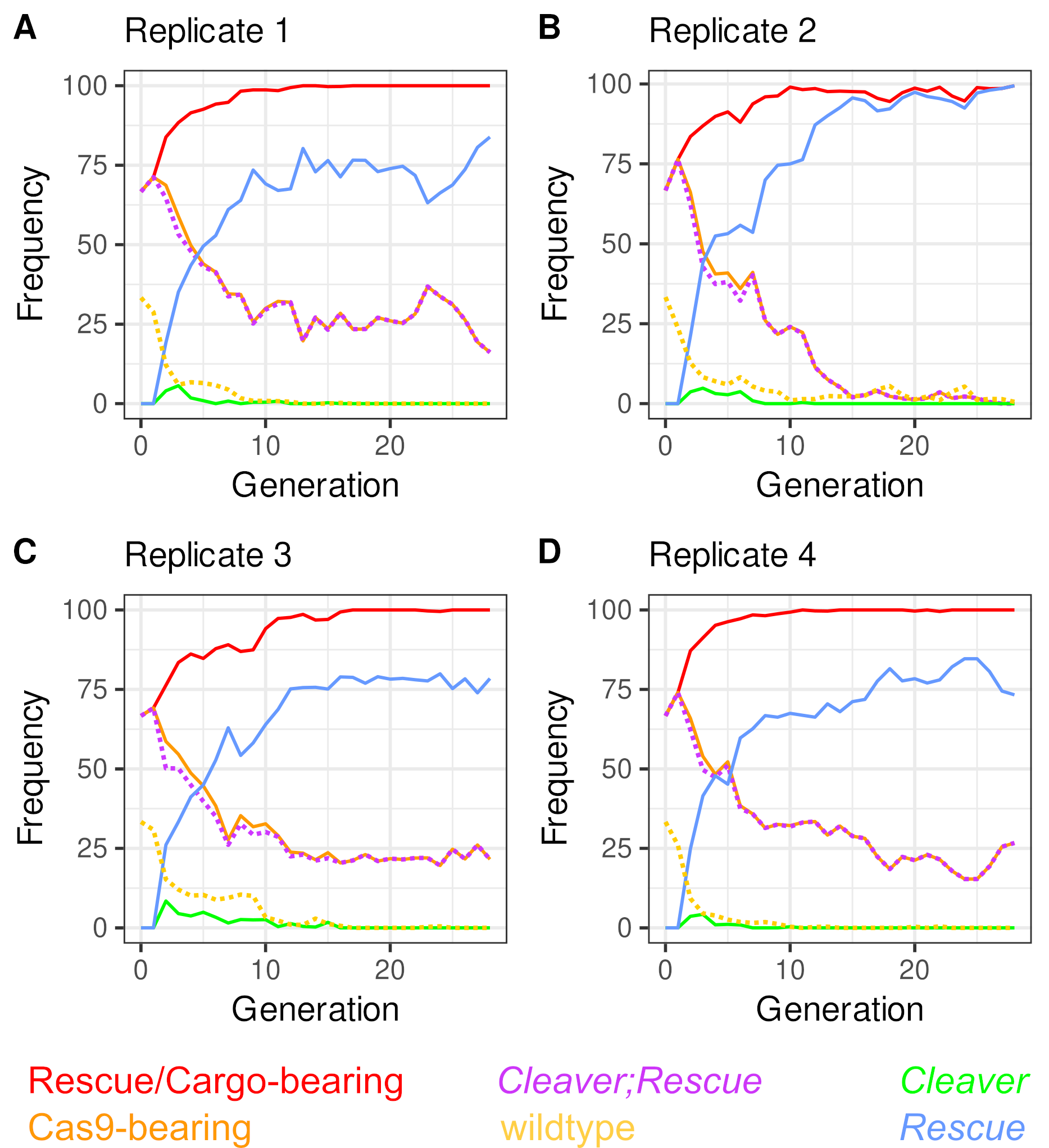

Supplement: S11 Fig — Legend on top of panels with Rescue/Cargo-bearing in red, Cas9-bearing in orange, Cleaver/Rescue in violet, Cleaver-only in green, Rescue-only in blue, and WT in yellow. (A-D) Replicates A-D. WT and Cleaver;Rescue in dotted lines for visibility. (TIFF) [file pgen.1009385.s011.tiff]
