# Supplementary material for: Split versions of Cleave and Rescue selfish genetic elements for measured self limiting gene drive
Source: PLoS Genet. 2021 Feb 18;17(2):e1009385. doi: 10.1371/journal.pgen.1009385 (PMC7951863; doi:10.1371/journal.pgen.1009385)
Supplement: S1 Table — Shown are the genotype frequencies in the offspring of a cross between heterozygous Cleaver/+;Rescuetko/+ virgins and w1118 males. All of the offspring carried the dominant td-tomato marker of Rescuetko. (PDF) [file pgen.1009385.s012.pdf]

S1 Table. Cleavage rates to LOF in females. Shown are the genotype frequencies in the offspring of a cross between heterozygous *Cleaver/+;Rescue*^{tko}/+ virgins and w^{1118} males. All of the offspring carried the dominant td-tomato marker of *Rescue*^{tko}.

Bottle	*Rescue*-bearing	WT	freq *Rescue* (%)
A	507	0	100
B	345	0	100
C	636	0	100
D	720	0	100
E	424	0	100
F	461	0	100
**TOTAL**	**3093**	**0**	**>99.97**
