# Supplementary material for: Split versions of Cleave and Rescue selfish genetic elements for measured self limiting gene drive
Source: PLoS Genet. 2021 Feb 18;17(2):e1009385. doi: 10.1371/journal.pgen.1009385 (PMC7951863; doi:10.1371/journal.pgen.1009385)
Supplement: S2 Table — We measured allele frequencies of the drive elements by outcrossing 100 males from the different drive replicates at generation 25 to w1118 virgins and scored the offspring for their respective markers. C = Cleaver (Cas9, 3xP3-td-tomato), R = Rescuetko (Rescue, opie-tomato), + = WT. Examples of how scoring was performed are in S1 Data (allele frequencies). (PDF) [file pgen.1009385.s013.pdf]

S2 Table. Allele frequencies in the drive populations at generation 25. We measured allele frequencies of the drive elements by outcrossing 100 males from the different drive replicates at generation 25 to w^{1118} virgins and scored the offspring for their respective markers. C= *Cleaver* (Cas9, *3xP3-td-tomato*), R= *Rescue*^{tko} (*Rescue*, opie-tomato), +=WT. Examples of how scoring was performed are in Data S1 (allele frequencies).

**Replicate**	**R/R**	**R/+**	**C/+;R/R**	**C/C;R/R**	**C/+;R/+**	**total alleles**	**Cleaver_freq (%)**	**Rescue_freq (%)**
A	37	19	17	4	3	160	17.5	86.3
B	58	30	0	0	0	176	0	83
C	43	18	16	0	2	158	11.4	87.3
D	46	20	14	0	1	162	9.3	87
